# Trans-omic analysis reveals obesity-associated dysregulation of inter-organ metabolic cycles between the liver and skeletal muscle

**DOI:** 10.1016/j.isci.2021.102217

**Published:** 2021-02-20

**Authors:** Riku Egami, Toshiya Kokaji, Atsushi Hatano, Katsuyuki Yugi, Miki Eto, Keigo Morita, Satoshi Ohno, Masashi Fujii, Ken-ichi Hironaka, Saori Uematsu, Akira Terakawa, Yunfan Bai, Yifei Pan, Takaho Tsuchiya, Haruka Ozaki, Hiroshi Inoue, Shinsuke Uda, Hiroyuki Kubota, Yutaka Suzuki, Masaki Matsumoto, Keiichi I. Nakayama, Akiyoshi Hirayama, Tomoyoshi Soga, Shinya Kuroda

**Affiliations:** 1 Department of Computational Biology and Medical Sciences, Graduate School of Frontier Sciences, University of Tokyo, 5-1-5 Kashiwanoha, Kashiwa, Chiba 277-8562, Japan; 2 Department of Biological Sciences, Graduate School of Science, University of Tokyo, 7-3-1 Hongo, Bunkyo-ku, Tokyo 113-0033, Japan; 3 Laboratory for Integrated Cellular Systems, RIKEN Center for Integrative Medical Sciences, 1-7-22 Suehiro-cho, Tsurumi-ku, Yokohama, Kanagawa 230-0045, Japan; 4 Department of Omics and Systems Biology, Graduate School of Medical and Dental Sciences, Niigata University, 757 Ichibancho, Asahimachi-dori, Chuo-ku, Niigata City, Niigata 951-8510, Japan; 5 Institute for Advanced Biosciences, Keio University, Fujisawa, 252-8520, Japan; 6 PRESTO, Japan Science and Technology Agency, 1-7-22 Suehiro-cho, Tsurumi-ku, Yokohama, Kanagawa 230-0045, Japan; 7 Molecular Genetics Research Laboratory, Graduate School of Science, University of Tokyo, 7-3-1 Hongo, Bunkyo-ku, Tokyo 113-0033, Japan; 8 Department of Mathematical and Life Sciences, Graduate School of Integrated Sciences for Life, Hiroshima University, 1-3-1 Kagamiyama, Higashi-hiroshima City, Hiroshima, 739-8526, Japan; 9 Bioinformatics Laboratory, Faculty of Medicine, University of Tsukuba, 1-1-1 Tennodai, Tsukuba, Ibaraki, 305-8575, Japan; 10 Center for Artificial Intelligence Research, University of Tsukuba, 1-1-1 Tennodai, Tsukuba, Ibaraki, 305-8577, Japan; 11 Metabolism and Nutrition Research Unit, Institute for Frontier Science Initiative, Kanazawa University, 13-1 Takaramachi, Kanazawa, Ishikawa, 920-8641, Japan; 12 Division of Integrated Omics, Research Center for Transomics Medicine, Medical Institute of Bioregulation, Kyushu University, 3-1-1 Maidashi, Higashi-ku, Fukuoka 812-8582, Japan; 13 Department of Molecular and Cellular Biology, Medical Institute of Bioregulation, Kyushu University, 3-1-1 Maidashi, Higashi-ku, Fukuoka 812-8582, Japan; 14 Institute for Advanced Biosciences, Keio University, 246-2 Mizukami, Kakuganji, Tsuruoka, Yamagata, 997-0052, Japan; 15 Core Research for Evolutional Science and Technology (CREST), Japan Science and Technology Agency, Bunkyo-ku, Tokyo 113-0033, Japan

**Keywords:** Biological Sciences, Endocrinology, Systems Biology, Omics, Proteomics, Metabolomics, Transcriptomic

## Abstract

Systemic metabolic homeostasis is regulated by inter-organ metabolic cycles involving multiple organs. Obesity impairs inter-organ metabolic cycles, resulting in metabolic diseases. The systemic landscape of dysregulated inter-organ metabolic cycles in obesity has yet to be explored. Here, we measured the transcriptome, proteome, and metabolome in the liver and skeletal muscle and the metabolome in blood of fasted wild-type and leptin-deficient obese (*ob*/*ob*) mice, identifying components with differential abundance and differential regulation in *ob*/*ob* mice. By constructing and evaluating the trans-omic network controlling the differences in metabolic reactions between fasted wild-type and *ob*/*ob* mice, we provided potential mechanisms of the obesity-associated dysfunctions of metabolic cycles between liver and skeletal muscle involving glucose-alanine, glucose-lactate, and ketone bodies. Our study revealed obesity-associated systemic pathological mechanisms of dysfunction of inter-organ metabolic cycles.

## Highlights

• Multi-omic data in liver and skeletal muscle of WT and *ob*/*ob* mice were measured
• We developed the trans-omic network of differentially regulated metabolic reactions
• Dysregulation of inter-organ metabolic cycles associated with obesity was revealed

## Graphical abstract

**Figure:**
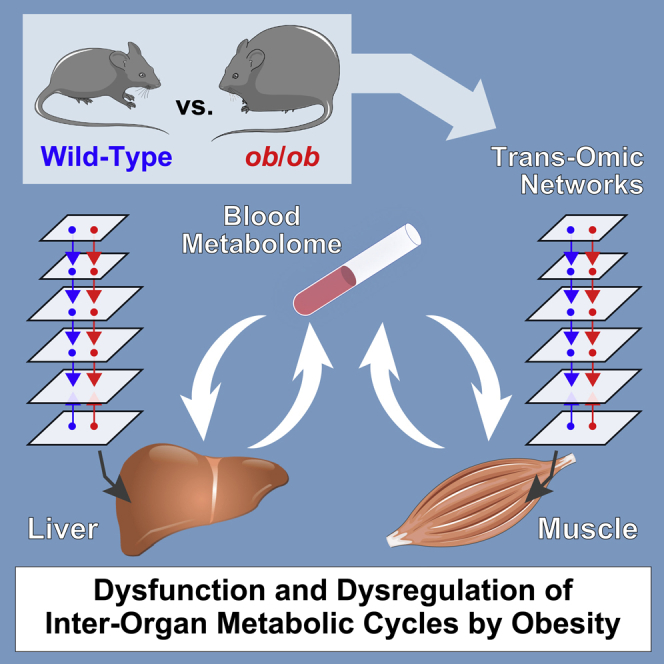

## Introduction

Systemic metabolic homeostasis is regulated not only by metabolism in each organ alone but also by inter-organ metabolic cycles, such as those between the liver and skeletal muscle, in mammals (Castillo-Armengol et al., 2019; Gancheva et al., 2018). Various metabolites produced by these organs are released into the circulation and affect the metabolism of other organs. Especially, during the fasting period, glucose and ketone bodies generated in the liver serve as essential energy sources for extrahepatic organs (Lehninger et al., 2005). On the other hand, lactate and alanine from the extrahepatic organs, like the skeletal muscle, work as substrates of glucose generation (gluconeogenesis) in the liver, which are known as Cori's cycle and glucose-alanine cycle, respectively (Cori and Cori, 1929; Felig, 1973). However, the detailed regulatory mechanisms controlling these inter-organ metabolic cycles are not fully understood.

Obesity impairs metabolic regulation of multiple organs, including the liver and skeletal muscle, leading to the dysfunction of the inter-organ metabolic cycle and subsequently to metabolic disorders, such as type 2 diabetes mellitus (Gancheva et al., 2018; Samuel and Shulman, 2016). Obesity perturbs metabolic regulation through changes in hormones, metabolites, and metabolic enzymes in the liver and skeletal muscle, leading to systemic metabolic dysfunction. Insulin resistance that is associated with obesity represents a well-characterized factor contributing to metabolic dysfunction (Kahn et al., 2006; Roden and Shulman, 2019); however, the effect of obesity on inter-organ metabolic cycles—the glucose-alanine cycle, the glucose-lactate cycle, and ketone body metabolism—have yet to be explored.

The metabolic network is complex, involving many diverse substrates, products, and intermediates in the chemical reactions of metabolism, denoted here as metabolic reactions, and the metabolic enzymes that mediate the reactions. Many metabolic enzymes are regulated not only through availability of substrates but also through feedback regulation by the products or through allosteric mechanisms. In addition, metabolic enzymes are regulated at the level of gene expression and through posttranslational modifications. Thus, obesity-associated dysregulation of metabolic networks in various organs is due to changes in complex networks with many forms of regulation (Lusis et al., 2008; Muoio and Newgard, 2008; Roden and Shulman, 2019). Hence, to comprehensively evaluate the effects of obesity on intracellular regulatory mechanisms requires investigation and integration of omic data of multiple types (transcriptomic, proteomic, and metabolomic) and from multiple organs. Few, if any, studies have taken such an integrated, multi-organ approach.

To effectively model regulation of metabolism requires not only integration of transcriptomic, proteomic, and metabolomic data but also using bioinformatics to link these datasets together through regulation. Transcriptional changes need to be connected to transcription factors (TFs) and posttranslational changes need to be connected to the enzymes mediating those modifications, such as connecting kinases to phosphorylation events. Metabolite regulation of metabolic enzymes requires linking such metabolites to relevant enzymes. Hence, metabolic reactions are regulated by an integrated network consisting of metabolites as substrates and products as well as allosteric regulators and their activation status, metabolic enzymes and their activation status, TFs and their activation status, and signaling molecules, such as kinases. Previously, we investigated the regulatory network controlling metabolic reactions by integrating simultaneous measurements of the amounts of metabolites, the expression of and the activation status of metabolic enzymes, and the amount and the activation status of signaling molecules into a multi-layered trans-omic network that also includes inferred TFs and metabolite-mediated regulation of metabolic reactions (Kawata et al., 2018; Kokaji et al., 2020; Yugi and Kuroda, 2017, 2018; Yugi et al., 2014, 2019). To date, we have used analysis of trans-omic network to establish a regulatory network of metabolic reactions changed by insulin stimulation in cultured cells and that of metabolic reactions responded to glucose administration in the livers of healthy and obese mice (Kokaji et al., 2020). Soltis et al. reported a molecular interaction network from molecules changed by high-fat-diet-associated obesity in multiple omic data but only of the liver (Soltis et al., 2017). Thus, the available studies have only evaluated either cultured cells or a single organ.

Because obesity impairs metabolic homeostasis in various organs, such as the liver and skeletal muscle, and inter-organ metabolic cycles between them through the blood, it is necessary to generate a trans-omic network that includes multi-omic data from the relevant organs and data from blood samples. The leptin-deficient obese (*ob*/*ob*) mice are a widely used model of obesity and insulin resistance, because these mice become profoundly obese by overeating because of deficiency of the anorexigenic hormone leptin (Ingalls et al., 1950; Lindström, 2007). Here, we constructed trans-omic networks for the liver and skeletal muscle by comparing data obtained from fasted wild-type (WT) and *ob*/*ob* mice. Using these trans-omic networks, we provided potential mechanisms of the dysfunctions of the glucose-alanine, glucose-lactate, and ketone body cycles between the liver and skeletal muscle, which characterize the dysfunctional systemic metabolism in *ob*/*ob* mice.

## Results

### Overview of the approach for constructing trans-omic networks from WT and *ob*/*ob* mice

**Figure 1:**
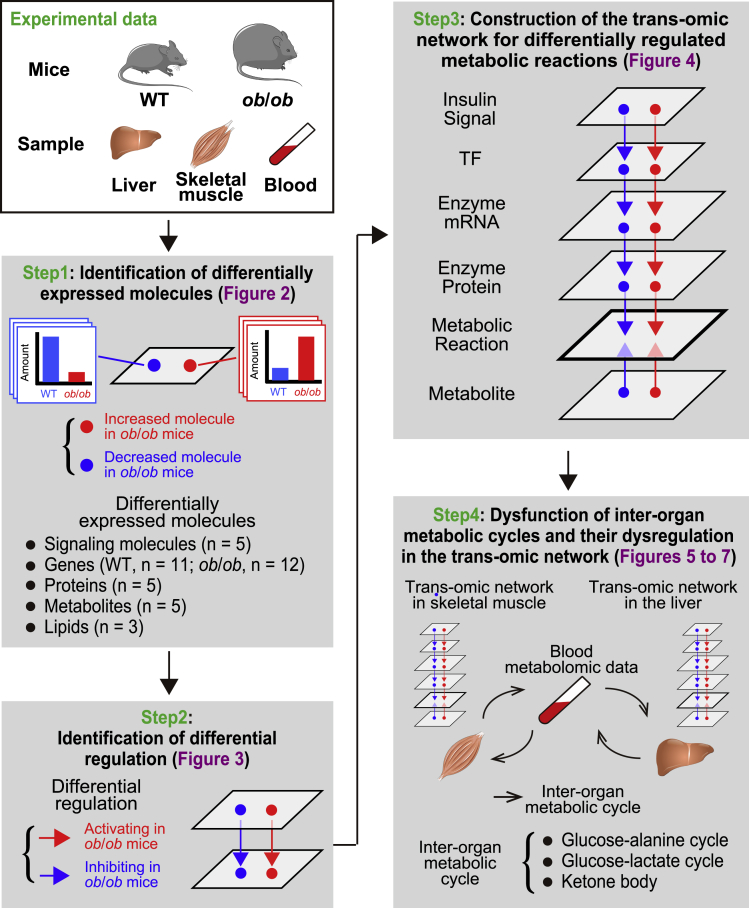
Overview of the approach for constructing trans-omic networks from WT and *ob*/*ob* mice We used WT and *ob*/*ob* mice and collected the liver, skeletal muscle, and the blood from 16-h-fasted mice. We measured signaling molecules, genes, proteins, metabolites, and lipids in the liver and skeletal muscle and identified differentially expressed molecules that significantly increased or decreased in *ob*/*ob* mice compared with WT mice in each omic layer (Step 1). We also measured metabolites in the blood. We identified the regulations connecting regulating differentially expressed molecules with regulated differentially expressed molecules using bioinformatic methods and multiple databases and defined those as “differential regulations” (Step 2). The differential regulations are classified into either activating regulation in *ob*/*ob* mice compared with WT mice (red arrow) or inhibiting regulation in *ob*/*ob* mice compared with WT mice (blue arrow). By integrating differentially expressed molecules and differential regulations that are involved in “metabolic reactions,” we constructed the trans-omic network for differentially regulated metabolic reactions in the liver and skeletal muscle (Step 3). Together with the blood metabolomic data, we examined dysfunction of inter-organ metabolic cycles and their dysregulation in the trans-omic network in the liver and skeletal muscle of *ob*/*ob* mice (Step 4). We used the following number of mouse replicates for each analysis: n = 5 both in WT and *ob*/*ob* mice for the identification of phosphorylated molecules, n = 11 (WT mice) and n = 12 (*ob*/*ob* mice) for transcriptomics, n = 5 both in WT and *ob*/*ob* mice for proteomics, n = 5 both in WT and *ob*/*ob* mice for metabolomics, and n = 3 both in WT and *ob*/*ob* mice for lipidomics.

We constructed trans-omic networks for differentially regulated metabolic reactions in liver and skeletal muscle between WT and *ob*/*ob* mice. We measured the amount and phosphorylation status of signaling molecules, global gene expression, global protein expression, and the amounts of metabolites and lipids in liver and skeletal muscle, along with the amounts of metabolites in the blood, of WT and *ob*/*ob* mice. We then selected the data relevant to metabolic reactions from the transcriptomic and proteomic datasets. By integrating these data, we constructed trans-omic networks for differentially regulated metabolic reactions in liver and skeletal muscle of *ob*/*ob* mice compared with WT mice (Figure 1).

We used 10-week-old WT and *ob*/*ob* mice that had fasted for 16 h. We collected liver, skeletal (gastrocnemius) muscle, and blood from the mice. Some of the liver and blood data were those reported in our previous study (Kokaji et al., 2020). The skeletal muscle data were obtained in this study and were from the same mice that were used to obtain the liver and blood data (Kokaji et al., 2020). Fasting glucose and insulin concentrations in the blood of *ob*/*ob* mice were significantly higher than those of WT mice, indicating that *ob*/*ob* mice exhibit the expected hyperglycemia and hyperinsulinemia (Figure S1).

To identify molecules (phosphorylated proteins, transcripts, proteins, metabolites, and lipids) that were differentially abundant between WT and *ob*/*ob* mice, we set the FDR-adjusted p value (q value) of less than 0.1 (Figure 1: Step1). We classified the differentially expressed molecules as either increased or decreased in *ob*/*ob* mice relative to their abundance in WT mice. We identified the regulatory connections for differentially expressed molecules using multiple databases and defined those as “differential regulations” (Figure 1: Step2). We classified the differential regulations as either activating or inhibiting in *ob*/*ob* mice compared with WT mice. By integrating the differentially expressed molecules and the differential regulations that are involved in “metabolic reaction,” we constructed a liver trans-omic network and a skeletal muscle trans-omic network for differentially regulated metabolic reactions (Figure 1: Step3). We grouped differentially expressed molecules from each type of data into layers connected by the differential regulations. The top layer represented signaling input from insulin (Insulin Signal) connected to a TF layer connected to layer representing the differentially expressed genes encoding metabolic enzymes (Enzyme mRNA) connected to layer representing the differentially expressed metabolic enzymes (Enzyme Protein) connected to a metabolic reactions layer (Metabolic Reaction). The Metabolic Reaction layer included regulatory input from both the Enzyme Protein layer and a metabolites (Metabolite) layer through allosteric regulation by metabolites and by changes in the amounts of substrates or products. Together with the blood metabolomic data, we used the trans-omic networks to identify dysfunction and dysregulation of inter-organ metabolic cycles between liver and skeletal muscle of *ob*/*ob* mice (Figure 1: Step4).

### Identification of differentially expressed molecules in liver and skeletal muscle of *ob*/*ob* mice

**Figure 2:**
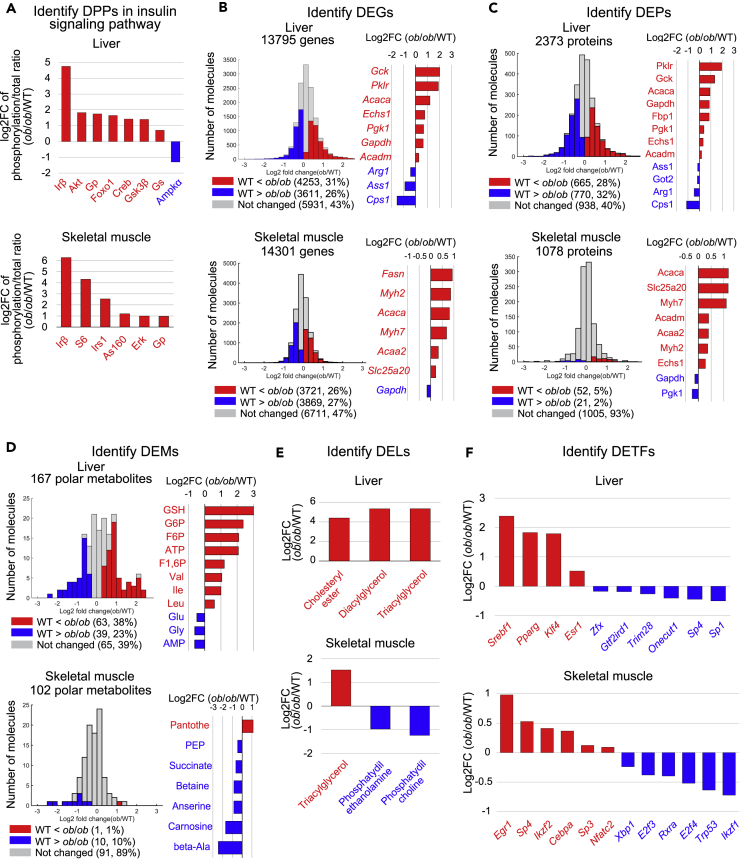
Identification of differentially expressed molecules in liver and skeletal muscle of *ob*/*ob* mice (A) The log_2_ fold changes of differentially phosphorylated proteins (DPPs) whose ratio significantly changed between WT and *ob*/*ob* mice. Red, the increased DPPs; blue, the decreased DPPs. See also Figure S2 and Table S1. (B) Histogram of log_2_ fold changes of the expression of genes between WT and *ob*/*ob* mice (left). Red, the increased differentially expressed genes (DEGs); blue, the decreased DEGs. The log_2_ fold changes of the indicated DEGs (right). See also Table S2. (C) Histogram of log_2_ fold changes of the expression of proteins between WT and *ob*/*ob* mice (left). Red, the increased differentially expressed proteins (DEPs); blue, the decreased DEPs. The log_2_ fold changes of the indicated DEPs (right). See also Table S3. (D) Histogram of log_2_ fold changes of the amounts of polar metabolites between WT and *ob*/*ob* mice (left). Red, the increased differentially expressed metabolites (DEMs); blue, the decreased DEMs. The log_2_ fold changes of the indicated DEMs (Right). See also Table S4. (E) The log_2_ fold changes of the amounts of the increased differentially expressed lipids (DELs) in the liver between WT and *ob*/*ob* mice. See also Table S5. (F) The log_2_ fold changes of gene expression of inferred TFs, which are the DETFs. Data are shown as the mean of mice replicate (number indicated in transparent methods). See also Figure S13 and Table S6. See Table S1 for the unabbreviated names of the phosphorylated molecules. Metabolites are abbreviated as follows: ATP, Adenosine triphosphate; GSH, Glutathione; G6P, Glucose-1-phosphate; F6P, Fructose-6-phosphate; F1,6P, Fructose-1,6-bisphosphate; PEP, Phosphoenolpyruvate; Pantothe, Pantothenate; beta-Ala, beta-alanine. Note that error bars are not provided because the fold change of averaged values of WT and *ob*/*ob* mice were used.

Using livers and skeletal muscle from fasted WT and *ob*/*ob* mice, we measured the total amount and phosphorylation of 29 proteins involved in insulin signaling by western blotting (Figure S2; Table S1). We also calculated the ratio of phosphorylated to total protein for 14 paired molecules and identified those with a ratio of phosphorylated to total that was significantly different between WT and *ob*/*ob* mice as the differentially phosphorylated proteins (DPPs) (Figure 2A). The only increased DPP in both the liver and skeletal muscle of *ob*/*ob* mice was the beta subunit of the insulin receptor (Irβ), which is consistent with the hyperinsulinemia observed in *ob*/*ob* mice (Figure S1). The only decreased DPP was the energy-sensing kinase Ampkα in the liver, which is consistent with increased ATP and decreased AMP in *ob*/*ob* mice (see Figures 2D and S10A) (Hardie, 2011). Akt and Foxo1 were liver-specific increased DPPs in the liver, whereas Erk, S6, and As160 were skeletal-muscle-specific increased DPPs.

We measured the expression of 13,795 genes in the liver and 14,301 genes in skeletal muscle from fasted WT and *ob*/*ob* mice using RNA sequencing (RNA-seq) (Figure 2B; Table S2). We defined genes that were significantly different in *ob*/*ob* mice compared with WT mice as differentially expressed genes (DEGs). We identified 4,253 upregulated DEGs and 3,611 downregulated DEGs in liver of *ob*/*ob* mice and 3,721 upregulated DEGs and 3,869 downregulated DEGs in skeletal muscle (Figure 2B). In both the liver and skeletal muscle, we observed the upregulation of genes involved in lipid metabolism (*Acaca*). In contrast, the DEGs encoding proteins involved in glycolysis and gluconeogenesis (*Gck*, *Gapdh*, *Pgk1*, *Pklr*) were upregulated in the liver, whereas the DEG encoding proteins involved in glycolysis and gluconeogenesis (*Gapdh*) were downregulated in skeletal muscle.

Using iBAQ mass spectrometry (iBAQ-MS), we measured the expression of proteins in the liver and skeletal muscle from fasted WT and *ob*/*ob* mice. We defined the significantly different proteins in *ob*/*ob* mice compared with WT mice as differentially expressed proteins (DEPs) (Figure 2C; Table S3). Out of 2,373 proteins detected in liver, we identified 665 increased DEPs and 770 decreased DEPs in *ob*/*ob* mice; out of 1,078 proteins detected in skeletal muscle, we identified 52 increased DEPs and 21 decreased DEPs in *ob*/*ob* mice (Figure 2C). The increased DEPs in the liver of *ob*/*ob* mice included the metabolic enzymes in glycolysis/gluconeogenesis (Gck, Gapdh, Pgk1, Pklr), whereas the decreased DEPs in skeletal muscle of *ob*/*ob* mice included those in the glycolysis/gluconeogenesis (Gapdh, Pgk1).

Using capillary electrophoresis mass spectrometry (CE-MS), we measured the amounts of polar metabolites, including carbohydrates, amino acids, polar lipids, and nucleic acids, in the liver and skeletal muscle from fasted WT and *ob*/*ob* mice, and defined polar metabolites that significantly changed in *ob*/*ob* mice compared with WT mice as the differentially expressed metabolites (DEMs) (Figure 2D; Table S4). We detected 167 polar metabolites in liver and identified 63 increased and 39 decreased DEMs in *ob*/*ob* mice (Figure 2D). In the skeletal muscle, we detected 102 polar metabolites with only 1 that increased, pantothenate, and 10 that decreased in *ob*/*ob* mice (Figure 2D). The increased DEMs in the liver of *ob*/*ob* mice included the cofactors ATP and glutathione (GSH), intermediate metabolites of the glycolysis/gluconeogenesis (glucose-6-phosphate (G6P), fructose-6-phosphate (F6P), and fructose-1,6-bisphosphate (F1,6P)), and the branched-chain amino acids (BCAAs) (Val, Leu, and Ile), and the decreased DEMs included AMP. The decreased DEMs in skeletal muscle from *ob*/*ob* mice included several that are particularly abundant in muscle: succinate, phosphoenolpyruvate (PEP), some amino acids such as beta-alanine and betaine, and the antioxidants anserine and carnosine, which are abundant in muscle compared with other organs (Kohen et al., 1988; Boldyrev et al., 2013).

Using liquid chromatography mass spectrometry (LC-MS), we measured the amount of 14 lipids in liver and skeletal muscle of fasted WT and *ob*/*ob* mice and defined lipids that significantly changed in *ob*/*ob* mice compared with WT mice as the differentially expressed lipids (DELs) (Figure 2E and Table S5). We identified three increased DELs in the liver and no decreased DELs. In contrast, skeletal muscle had one increased DEL and ten decreased DELs. Triacylglycerol (TAG) increased in both liver and skeletal muscle of fasted *ob*/*ob* mice. The skeletal-muscle-specific decreased DELs included phosphatidyl ethanolamine (PE) and phosphatidyl choline (PC) in skeletal muscle (Figure 2E).

Gene expression is regulated by the TF. Here, we inferred the TFs that regulate the DEGs by the TF motif enrichment analysis using the TF binding motif database, TRANSFAC (Matys et al., 2006; Kel et al., 2003). In liver, we identified 7 TF motifs for the increased DEGs and 14 TF motifs for the decreased DEGs in *ob*/*ob* mice; in skeletal muscle, we identified 17 TF motifs for the increased DEGs and 16 TF motifs for the decreased DEGs (Table S6). Of the TFs corresponding to inferred TF motifs, we identified those included in the DEGs and defined them as the differentially expressed TFs (DETFs). In liver, the increased DETFs included *Srebf1*, *Pparg*, *Klf4*, and *Esr1*, whereas the decreased DETFs included *Sp1* and *Trim28* (Figure 2F; see also Figure S13). In skeletal muscle, the increased DETFs included *Cebpa*, *Sp4*, and *Egr1*, whereas the decreased DETFs included *Xbp1* and *Trp53* (Figure 2F). We identified the Foxo1 motif for the decreased DEGs (Table S6) and identified the pFoxo1 as an increased DPP (Figure 2A). We defined pFoxo1 as a differentially phosphorylated TF (DPTF). Together with the fact that transcriptional activity of Foxo1 decreased by phosphorylation-mediated nuclear export (Barthel et al., 2005), the transcriptional activity of Foxo1 is likely to be decreased in *ob*/*ob* mice. Hereafter, we defined the DETFs and the DPTF as differentially regulated TFs (DRTFs). The skeletal-muscle-specific increased DRTFs included *Egr1*. Given that Foxo1 and *Egr1* are downstream molecules of Akt (Barthel et al., 2005) and Erk (Thiel and Cibelli, 2002), respectively, these identified DRTFs are consistent with the liver-specific increased phosphorylation of Akt and skeletal-muscle-specific increased phosphorylation of Erk (Figure 2A).

### Identification of differential regulation in liver and skeletal muscle of *ob*/*ob* mice

**Figure 3:**
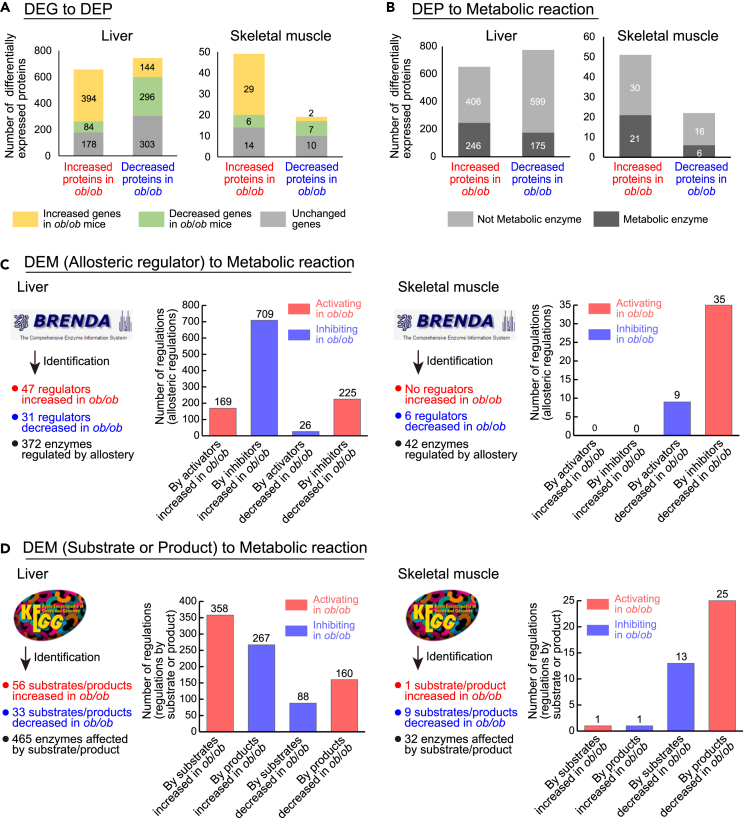
Identification of differential regulation in liver and skeletal muscle of *ob*/*ob* mice (A) The number of DEPs encoded by DEGs in the liver and skeletal muscle. The IDs of proteins and genes were unified based on Ensembl protein ID. See also Figure S3 and Table S7. (B) The number of metabolic enzymes among the DEPs in the liver and skeletal muscle. The IDs of proteins were unified based on Entrez. (C) The number of allosteric regulators of DEMs and metabolic enzymes regulated by those identified by the analysis using BRENDA and the number of allosteric regulations for metabolic enzymes. See also Figure S4. (D) The number of substrate or product of DEMs and metabolic enzymes regulated by those identified by the analysis using KEGG and the number of regulations by substrate or product for metabolic enzymes. See also Figure S4.

We identified the differential regulations connecting regulating differentially expressed molecules with regulated differentially expressed molecules. To identify differential regulations linking DPPs in the insulin signaling molecules to DRTFs, we searched KEGG (Kanehisa et al., 2017) for kinase-substrate relationships between the DPPs and DRTFs. The only differential regulations we found was from Akt, an increased DPP in liver, to Foxo1, an increased DRTF in liver (Figure 2A). The differential regulations between DRTFs and DEGs were determined based on the relationship between the DRTF and the change in expression of the DEG. To identify differential regulations from DEGs to DEPs, we examined the DEP and DEG datasets for protein-encoding gene pairs that showed consistent changes in gene expression and protein expression in *ob*/*ob* mice (Figure 3A; see also Figure S3 and Table S7). In liver, 394 increased DEPs (60% of the total 565 increased DEPs) had upregulated DEGs, and 296 decreased DEPs (40% of the total 743 decreased DEPs) had downregulated DEGs. In skeletal muscle, 29 increased DEPs (59% of total 49 increased DEPs) had correspondingly increased DEGs, whereas 7 decreased DEPs (37% of total 19 decreased DEPs) had decreased DEGs. These data suggested that, although more DEPs were regulated in liver than in skeletal muscle, similar proportions of DEPs were regulated at the level of gene expression in both liver and skeletal muscle in *ob*/*ob* mice.

To examine the expression of metabolic enzymes responsible for metabolic reactions, we identified metabolic enzymes among the DEPs and defined them as the differentially expressed metabolic enzymes (DEMEs). We identified 246 increased and 175 decreased DEMEs in the liver of *ob*/*ob* mice and 21 increased and 6 decreased DEMEs in skeletal muscle of *ob*/*ob* mice (Figure 3B).

The activity of metabolic reactions is regulated not only by the expression of metabolic enzymes but also through allosteric regulation by metabolites and by changes in the amounts of substrates or products. We identified allosteric regulations connecting DEMs to metabolic reactions using the database BRENDA (Jeske et al., 2019) (Figures 3C and S4A). The differential regulations from DEMs were classified as either activating or inhibiting and were determined by combining the direction of the change in a DEM (increased or decreased) and the direction of its regulatory effect on the reaction (activator or inhibitor) (Figure S4A). In liver, we identified 372 metabolic enzymes that were controlled by DEMs that are allosteric regulators and total 1,129 allosteric regulations to metabolic reactions, with AMP contributing most of the activating allosteric regulation and ATP and ADP inhibiting many reactions through allostery (Figures 3C and S4B). In skeletal muscle, we identified 42 metabolic enzymes that were controlled by DEMs, and both activating and inhibiting allosteric regulations were mediated by DEMs that decreased in *ob*/*ob* mice (Figures 3C and S4D). Among the DEMs, we also identified the substrates and products that can affect metabolic reactions using the database KEGG (Kanehisa et al., 2017) (Figures 3D and S4A). In liver, we identified 465 metabolic enzymes affected by DEMs that are substrates or products and 873 regulations between those enzymes and the DEMs that are substrates or products (Figures 3D and S4C). Some DEMs, including ATP, ADP, and NAD^+^, were involved in both allosteric regulations and regulations by substrate or product in the liver of *ob*/*ob* mice. In skeletal muscle, we identified 32 metabolic enzymes affected by changes of the substrates or products and 40 differential regulations by substrate or product DEMs (Figures 3D and S4E).

### Construction of trans-omic networks for differentially regulated metabolic reactions in liver and skeletal muscle of *ob*/*ob* mice

**Figure 4:**
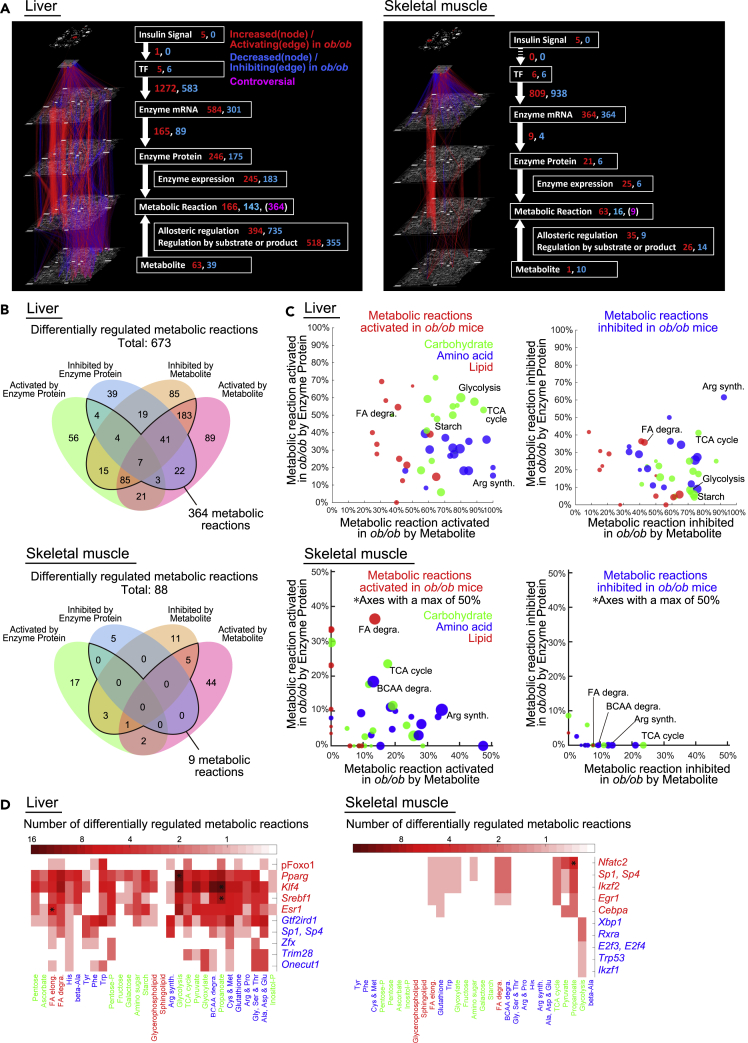
Construction of trans-omic networks for differentially regulated metabolic reactions in liver and skeletal muscle of *ob*/*ob* mice (A) The trans-omic network for differentially regulated metabolic reactions in the liver and skeletal muscle. Nodes and edges indicate differentially expressed molecules and differential regulations involved in metabolic reactions, respectively. Red, increased (nodes) or activating (edges) in *ob*/*ob* mice; blue, decreased (nodes) or inhibiting (edges) in *ob*/*ob* mice; magenta, controversial metabolic reactions had opposing regulatory input from Enzyme Protein layer and Metabolite layer. The numbers of differentially expressed molecules (nodes) and differential regulations (edges) are shown. (B) The number of differentially regulated metabolic reactions controlled by differentially expressed molecules in the Enzyme Protein layer or the Metabolite layer or both in the liver and skeletal muscle. (C) For each metabolic pathway in the liver and skeletal muscle, the percentage of activated (upper) or inhibited (lower) metabolic reactions by Metabolite (*x* axis) and by Enzyme Protein (*y* axis) are plotted. The size of the dots indicates the number of differentially regulated metabolic reactions in each metabolic pathway regulated either by Metabolite, Enzyme Protein, or both in each organ. The colors of the dots indicate the classes of metabolic pathway according to the KEGG database: carbohydrate (green), amino acid (blue), and lipid (red). (D) The number of differentially regulated metabolic reactions in each metabolic pathway (rows) regulated by each differentially regulated transcription factor (DRTF) (columns) in the liver and skeletal muscle. The sign ∗ indicates the significant associations (q value <0.01) between differentially regulated metabolic reactions in the metabolic pathway node and those regulated by the DRTF. The q value was calculated by one-tailed Fisher's exact test. See also Figures S5–S10.

To elucidate the regulatory networks controlling the differences in metabolic reactions in liver and skeletal muscle between fasted WT and *ob*/*ob* mice, we constructed trans-omic networks for differentially regulated metabolic reactions in both organs (Figure 4A). The trans-omic networks consisted of two parts, the differentially expressed molecules, which are the nodes in each layer (Figure S5, upper left), and the differential regulations, which are edges connecting nodes in different layers (Figure S5, upper right). The Enzyme mRNA layer contained DEGs for all metabolic enzymes in the pathways in “Metabolism” from the KEGG database (Kanehisa et al., 2017). The Enzyme Protein layer contained the DEMEs. The Metabolic Reaction layer contained the metabolic reactions directly regulated by the DEMEs or DEMs and defined them as the differentially regulated metabolic reactions. Some metabolic enzymes mediate more than one reaction; thus, the number of reactions exceeds the number of enzyme proteins. The final trans-omic networks contained the differentially expressed molecules organized in six layers: insulin signaling molecules in DPPs in the Insulin Signal layer, DRTFs in the TF layer, DEGs encoding metabolic enzymes in the Enzyme mRNA layer, DEMEs in the Enzyme Protein layer, differentially regulated metabolic reactions in the Metabolic Reaction layer, and DEMs in the Metabolite layer, with the differential regulations connecting the layers. We classified the differential regulations as either activating or inhibiting the downstream molecules or reactions in *ob*/*ob* mice by combining the direction of changes of differentially expressed molecules (increased or decreased) and the direction of regulation (activation or inhibition) (Figure S5 lower table). The trans-omic network included the differential regulations for various metabolic pathways: glycogen metabolism (Figure S6A), TCA cycle (Figure S6B), glycolysis/gluconeogenesis (see Figures 6A and 6B), fatty acid synthesis (Figure S7), fatty acid degradation (see Figures 7A and 7B), arginine synthesis (Figure S8), BCAA degradation (Figure S9), purine metabolism (Figure S10A), and pyrimidine metabolism (Figure S10B).

For liver, the resulting network included 673 metabolic reactions that were differentially regulated in fasted *ob*/*ob* mice and that had regulatory inputs from the Enzyme Protein layer and the Metabolite layer (Figure 4A, left). Of these 673 metabolic reactions, 364 reactions had opposing regulatory input from those two layers, thus we classified these as “controversial” (Figure 4B, upper). For all differential regulations except those involving allosteric regulation, the number of activating regulations exceeded the number of inhibiting regulations and the number of differentially expressed molecules that increased exceeded those that decreased except in the TF layer, which had equivalent numbers of DRTFs with increased and decreased activity.

For skeletal muscle, the trans-omic network was smaller with only 88 metabolic reactions that were differentially regulated in fasted *ob*/*ob* mice (Figure 4A, right). In contrast to the liver trans-omic network, there was no phosphorylation-mediated regulation from the Insulin Signal layer to the TF layer in skeletal muscle. The number of increased DEMEs was larger than that of decreased DEMEs in the Enzyme Protein layer, whereas the number of the increased DEMs was smaller than that of the decreased DEMs in the Metabolite layer. For all three types of regulation of enzyme activity—enzyme expression, allosteric regulation, and regulation by substrate or product—the number of activating regulations was larger than that of the inhibiting regulations in skeletal muscle of *ob*/*ob* mice (Figure 4A, right). We calculated the number of differentially regulated metabolic reactions regulated by changes in the Enzyme Protein or Metabolite layers (Figure 4B, lower). In contrast to liver with ∼54% of reactions having controversial opposing regulation, skeletal muscle metabolic reactions were more consistently regulated with only 9 of 88 (∼10%) reactions regulated in an opposing manner by changes in the Enzyme Protein and Metabolite layers in skeletal muscle.

To elucidate key mechanisms involved in the differences of metabolic reaction between WT mice and *ob*/*ob* mice in liver and skeletal muscle, we grouped metabolic reactions belonging to the same metabolic pathway and calculated the percentage of activated or inhibited metabolic reactions by Metabolite and by Enzyme Protein layers (Figure 4C). We focused on pathways representing carbohydrate, amino acid, and lipid metabolism. Nucleotide metabolism is separately described in supplemental information (see Figures S10A and S10B). In liver, several carbohydrate metabolic pathways, including glycolysis/gluconeogenesis, the starch (glycogen) metabolism, and TCA cycle, and several lipid metabolic pathways, including fatty acid degradation, had more activating regulations than inhibiting regulations by DEMEs (Figures 4C, S7A, and S7B; see also Figures 6A and 7A). This result suggests that these metabolic pathways are activated at the level of protein expression of metabolic enzymes in fasted *ob*/*ob* mice. In skeletal muscle, some metabolic pathways were activated by DEMEs, such as fatty acid degradation and BCAA degradation (Figures 4C and S9 lower; see also Figure 7B). In addition, in both organs, most amino acid metabolic pathways were regulated by DEMs in *ob*/*ob* mice. An exception was inhibition of arginine synthesis pathway in the liver, which involved a high percentage of metabolic reactions controlled by both DEMs and DEMEs (Figures 4C and S8 upper).

We also evaluated the number of metabolic reactions regulated by the increased and decreased DRTFs in the trans-omic networks for liver and skeletal muscle from *ob*/*ob* mice (Figure 4D). This analysis showed that increased DRTFs had a greater impact on the networks than decreased DRTFs. It also showed that across carbohydrate, lipid, and amino acid metabolism, liver showed more complex effects of changes in gene expression with many metabolic pathways having both input from increased and decreased DRTFs. In addition, in liver *Esr1* (*Estrogen receptor 1*) was significantly associated with fatty acid elongation, *Srebf1* (*Sterol regulatory element-binding protein 1*) and *Klf4* (*Kruppel-like factor 4*) were significantly associated with metabolism of the short fatty acid propanoate, and *Pparg* (*Peroxisome proliferator-activated receptor gamma*) was significantly associated with glycolysis/gluconeogenesis. In skeletal muscle, only *Nfatc2* (*Nuclear factor of activated T cells 2*) was significantly associated with any pathway and the association was with propanoate metabolism.

### Identification of differentially expressed metabolites in blood from *ob*/*ob* mice

**Figure 5:**
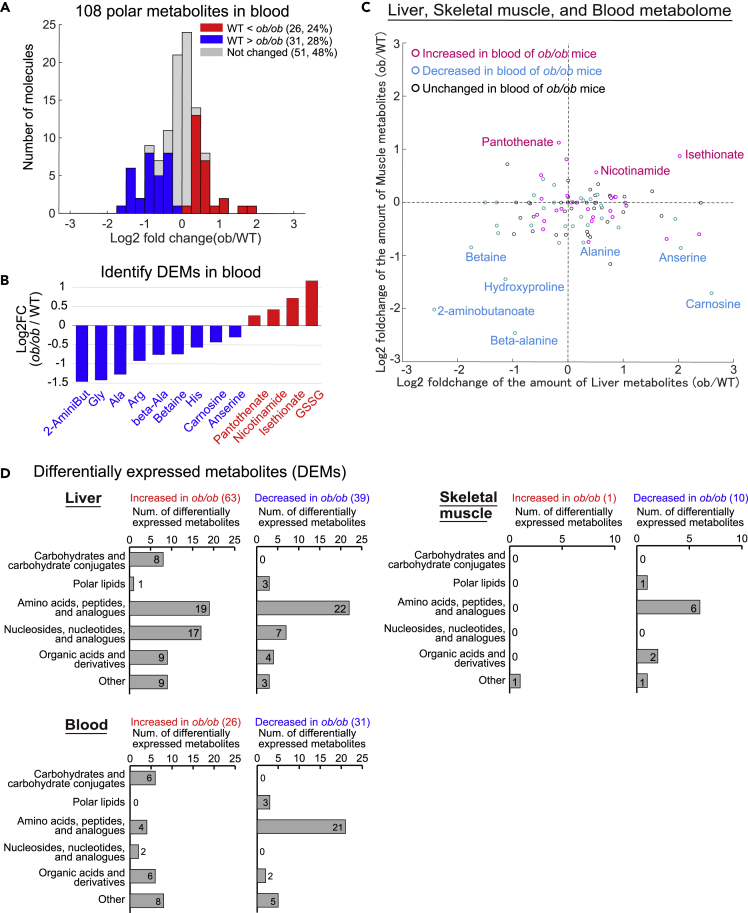
Identification of differentially expressed metabolites in blood from *ob*/*ob* mice (A and B) Metabolomic analysis using CE-MS in the blood of WT and *ob*/*ob* mice. Histogram showing distribution of log_2_ fold changes of the amount of blood polar metabolites between WT and *ob*/*ob* mice (A). The log_2_ fold changes of the amounts of the indicated DEMs in the blood between WT and *ob*/*ob* mice (B). Metabolites are abbreviated as follows: 2-AminoBut, 2-aminobutanoate; beta-Ala, beta-alanine; GSSG, Glutathione disulfide. Note that error bars are not provided because the fold change of averaged values of WT and *ob*/*ob* mice were used. See also Table S8. (C) The log_2_ fold changes of polar metabolites measured in the liver, skeletal muscle, and the blood between WT and *ob*/*ob* mice. The value of log_2_ fold changes of polar metabolites in the liver (*x* axis) and skeletal muscle (*y* axis) between WT and *ob*/*ob* mice are shown in scatterplot. The color of each node indicates whether the metabolite is increased DEMs (Magenta) or decreased DEMs (Cyan) in the blood of *ob*/*ob* mice. (D) The number of the DEMs classified by metabolite class of KEGG database in the liver, skeletal muscle, and blood. Note that we did not measure lipidome in blood. See also Figure S11.

The blood communicates changes in metabolism and metabolic requirements between organs through circulating metabolites. Through such circulation-mediated communication, the liver and skeletal muscle contribute to systemic metabolic homeostasis. Therefore, we analyzed polar metabolites in the blood of fasted WT and *ob*/*ob* mice and compared them with the changes in liver and skeletal muscle of *ob*/*ob* mice (Figures 5 and S11). We measured the amounts of 108 polar metabolites in the blood of WT and *ob*/*ob* mice, using CE-MS, and identified 26 increased and 31 decreased DEMs in the blood of *ob*/*ob* mice (Figure 5A; Table S8). The increased DEMs in the blood included glutathione disulfide (GSSG) and pantothenate. However, most of the decreased DEMs in the blood were amino acids (Ala, Gly, Arg, and His) and their derivatives (betaine, beta-alanine, anserine, and carnosine) (Figure 5B).

We compared the DEMs in the blood with those in the liver and those in skeletal muscle. We identified 11 increased and 11 decreased DEMs common in both blood and liver (Figure S11B) with a modest correlation (Pearson's correlation coefficient = 0.33) detected for these changes between fasted WT and *ob*/*ob* mice. Pantothenate, a core component of coenzyme A (CoA), was the only increased DEM in common between blood and skeletal muscle, and 6 decreased DEMs were common to blood and skeletal muscle (Figure S11B) with a modest correlation (Pearson's correlation coefficients = 0.43) detected for them. We examined the changes of metabolites across the three compartments—liver, skeletal muscle, and blood—between fasted WT and *ob*/*ob* mice (Figure 5C). This analysis revealed 4 metabolites—betaine, beta-alanine, hydroxyproline, and 2-aminobutanoate—significantly decreased in *ob*/*ob* mice across all three compartments. This analysis also revealed metabolites that selectively changed in each compartment. For example, alanine, the key factor in the glucose-alanine cycle, slightly increased in the liver but decreased in skeletal muscle and blood.

We also evaluated the DEMs by type of metabolite among the liver, skeletal muscle, and blood (Figure 5D). This comparison showed that amino acids, peptides, and related molecules showed predominantly decreases in blood and skeletal muscle but showed both increases and decreases in liver.

### Potential mechanisms of dysfunction of inter-organ metabolic cycles of glucose-alanine and glucose-lactate and their dysregulation in the trans-omic network in liver and skeletal muscle of *ob*/*ob* mice

**Figure 6:**
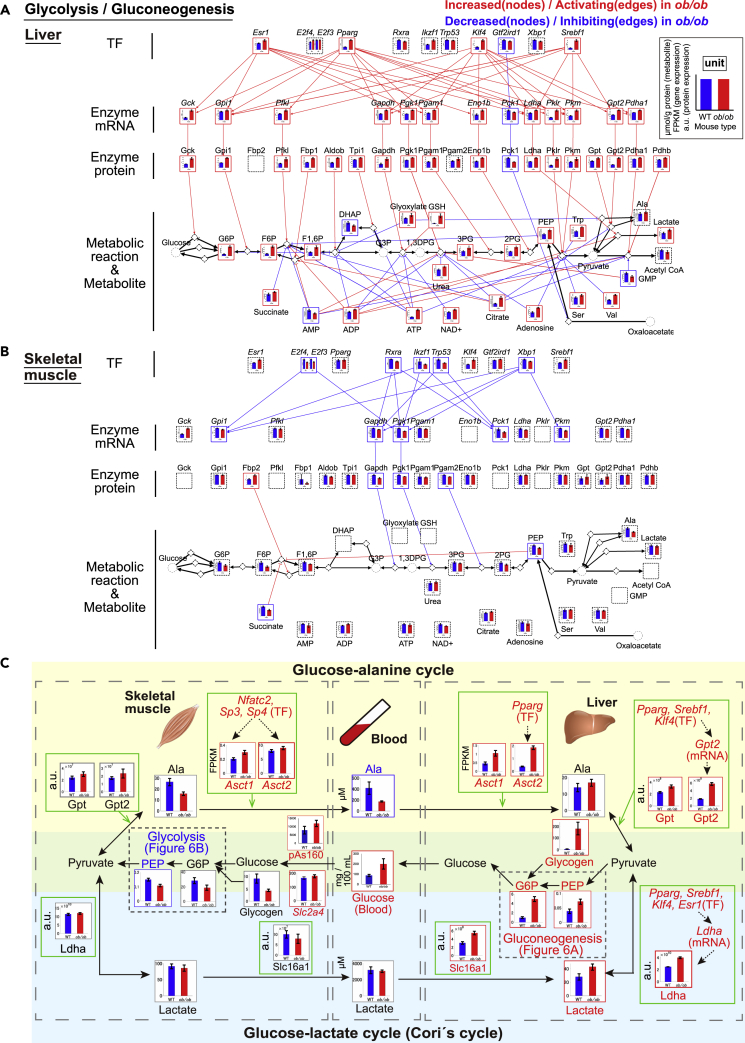
Potential mechanisms of dysfunction of inter-organ metabolic cycles of glucose-alanine and glucose-lactate and their dysregulation in trans-omic network in liver and skeletal muscle of *ob*/*ob* mice (A and B) The trans-omic subnetwork for differentially regulated metabolic reactions in glycolysis/gluconeogenesis in the liver (A) and skeletal muscle (B). The information for glycolysis/gluconeogenesis were obtained from “glycolysis/gluconeogenesis” (mmu00010) in the KEGG database, respectively (Kanehisa et al., 2017). The colors of the frames indicate differentially expressed molecules increased in *ob*/*ob* mice (red) and those decreased in *ob*/*ob* mice (blue). The significance of differences was tested by two-tailed Welch's t test for each measured protein and polar metabolite and by the edgeR package with default parameters for each measured gene (see also transparent methods). Differentially expressed molecules were molecules with a q value less than 0.1 and were classified as increased or decreased differentially expressed molecules. The q values were calculated by Storey's procedure for gene expression, protein expression, and polar metabolite. The dashed black frames showed molecules that were not differentially expressed molecules or undetected molecules in the indicated organ. Diamond nodes indicate metabolic reactions. The inter-layered red and blue edges indicate differential regulations: activating regulation in *ob*/*ob* mice (red) and inhibiting regulation in *ob*/*ob* mice (blue). We classified the differential regulations as either activating or inhibiting the downstream molecules in *ob*/*ob* mice by combining the direction of changes of regulating differentially expressed molecules (increased or decreased) and the direction of regulation (activation or inhibition). When the regulation between regulating upstream differentially expressed molecules and regulated downstream differentially expressed molecules is consistent, these regulations are displayed in the network as differential regulations. From Metabolite to Metabolic reaction, only allosteric regulations are colored. Black edges indicate the relationship between metabolic reactions and its substrate/product. The reversibility of metabolic reactions was obtained from the KEGG database. See also Figures S12A and S12B. (C) A summarized diagram of glucose-alanine (yellow highlighted) and glucose-lactate (light blue highlighted) cycles between liver and skeletal muscle via the blood of WT and *ob*/*ob* mice. The black edges represent the flow of metabolites. Green frames represent the regulation of the expression of indicated metabolic enzymes or transporters in the liver and skeletal muscle. The dotted arrows in the green frames are differential regulations in each organ. Red and blue frames indicate the increased differentially expressed molecules in *ob*/*ob* mice (red) and the decreased those in *ob*/*ob* mice (blue), respectively. Molecules with gray frames did not show the significant differences between WT and *ob*/*ob* mice. The significance of the differences in indicated molecules was determined in the same statistical methods described in Figures 6A and 6B. Bar plots of measured molecules in WT and *ob*/*ob* mice are shown for corresponding nodes as the means and standard error of the means (SEMs) of mice replicate (number indicated in transparent methods).

**Figure 7:**
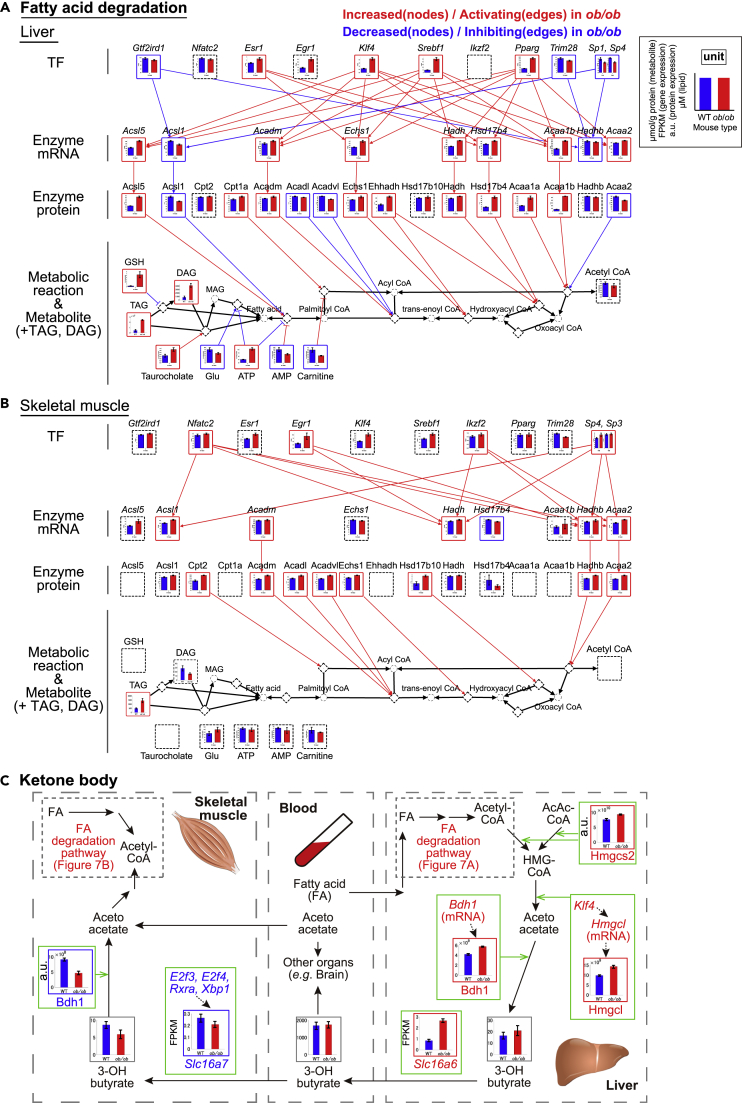
Potential mechanisms of dysfunction of inter-organ metabolic cycles of ketone body and their dysregulation in trans-omic network in liver and skeletal muscle of *ob*/*ob* mice (A and B) The trans-omic subnetwork for differentially regulated metabolic reactions in fatty acid degradation in the liver (A) and skeletal muscle (B). The information for fatty acid degradation were obtained from “fatty acid degradation” (mmu00071) and “glycerolipid metabolism” (mmu00561) in the KEGG database. Bar plots of measured molecules in WT and *ob*/*ob* mice are shown for corresponding nodes as the means and standard error of the means (SEMs) of mice replicate (number indicated in transparent methods). The definitions of color, edges, arrows, and frames in Figures 7A and 7B are the same as in Figures 6A and 6B, except lipidome data. In the lipidomic analysis, the significance of differences was tested by a two-tailed Welch's t test for each measured lipid (see also transparent methods). TAG and DAG in the liver and TAG in the skeletal muscle were differentially expressed lipids (DELs) with a q value less than 0.1 and were identified as the increased DELs. The q values for lipid were calculated by Benjamini–Hochberg procedure. See also Figures S12C and S12D. (C) A summarized diagram of ketone body cycle between the liver and skeletal muscle via the blood of WT and *ob*/*ob* mice. The definitions of color, edges, arrows, and frames in Figure 7C are the same as in Figure 6C. Bar plots of measured molecules in WT and *ob*/*ob* mice are shown for corresponding nodes as the means and SEMs of mice replicate (number indicated in transparent methods).

Because there is limited understanding of how obesity affects the main cycles between liver and skeletal muscle metabolism through the blood, we generated inter-organ metabolic networks for three cycles: the glucose-alanine cycle, the glucose-lactate cycle, and the ketone body cycle. Liver provides gluconeogenesis activity, whereas skeletal muscle consumes glucose through glycolysis. These processes are linked through two cycles (Dashty, 2013; Sarabhai and Roden, 2019). Alanine is transported to the liver and converted into glucose, which is then released into the circulation and taken up by skeletal muscle. This cycle is the glucose-alanine cycle (Felig, 1973). Skeletal muscle converts glucose to lactate, which is then transported to the liver for conversion to glucose. This cycle is called the glucose-lactate cycle (Cori's cycle) (Cori and Cori, 1929). When glucose is limiting, ketone bodies provide a source of energy from fatty acid degradation (Cahill et al., 1966). Therefore, we explored potential mechanisms of how dysregulation of the liver and skeletal muscle trans-omic subnetworks of glycolysis and gluconeogenesis for glucose-alanine and glucose-lactate cycles (Figure 6) and of fatty acid degradation for ketone body cycle (Figure 7) in fasted *ob*/*ob* mice affected these three metabolic cycles.

To investigate glucose-alanine and glucose-lactate cycles between liver and skeletal muscle, we extracted the trans-omic subnetwork of glycolysis/gluconeogenesis pathway from the trans-omic networks in the liver and skeletal muscle according to information of “glycolysis/gluconeogenesis” (mmu00010) in the KEGG database (Kanehisa et al., 2017). Most of the intermediate metabolites in the glycolysis/gluconeogenesis pathway in the liver, such as G6P, F6P, F1,6P, 3-phosphoglycerate (3PG), 2-phosphoglycerate (2PG), and PEP, were the increased DEMs in *ob*/*ob* mice except dihydroxyacetone phosphate (DHAP) (Figure 6A). Many metabolic enzymes were increased DEMEs in *ob*/*ob* mice (17 proteins: 94% of total 18 DEMEs), whereas the only decreased DEME was Pck1 (Figures 6A and S12A). The expression of metabolic enzymes specific to glycolysis, including rate-limiting enzymes, such as Gck and Pfkl, increased in *ob*/*ob* mice, whose genes were also upregulated by the increased DRTFs *Srebf1*, *Pparg*, and *Esr1*. The expression of metabolic enzymes specific to gluconeogenesis, including a rate-limiting enzyme Fbp1, increased in *ob*/*ob* mice. The expression of metabolic enzymes for overall glycolysis and gluconeogenesis pathways, including Gpi1, Gapdh, and Pgk1, increased in *ob*/*ob* mice. However, these enzymes are not rate-limiting enzymes and may not have large contributions to the regulation of glycolysis and gluconeogenesis. Taken together, these results suggest that both glycolysis and gluconeogenesis pathways are activated by the DEMEs in liver of *ob*/*ob* mice. Because it has been shown that gluconeogenesis is activated in liver of fasted obese human, *ob*/*ob* mice, and high-fat diet-fed mice (Turner et al., 2005; Basu et al., 2005; Satapati et al., 2012), gluconeogenesis is likely to be relatively more dominant than glycolysis in liver of fasted *ob*/*ob* mice. Besides, it has been reported to increase the blood glucose level in fasted *db*/*db* mice, a leptin receptor-deficient mice (Clementi et al., 2011; Liu et al., 2012), and high-fat diet-fed mice more than in wild-type mice and standard-chow-diet-fed mice (Soto et al., 2018), respectively, by intraperitoneal pyruvate administration. These results also support our interpretation that gluconeogenesis is more dominant than glycolysis. In contrast to activating regulation by the DEMEs, majority of allosteric regulation were inhibiting regulation in *ob*/*ob* mice (glycolysis specific: 3 activating and 13 inhibiting regulations, gluconeogenesis specific: 2 activating regulations, common for glycolysis and gluconeogenesis: 4 activating and 21 inhibiting regulations), most of which are mediated by the increased DEMs (Figures 6A and S12A). The activating regulations by the DEMEs and the inhibiting allosteric regulations by the increased DEMs counteract in liver of *ob*/*ob* mice, suggesting that in *ob*/*ob* mice, the DEMEs activate their targets, whereas the DEMs inhibit their targets.

In skeletal muscle of *ob*/*ob* mice, PEP was the only decreased DEM (Figure 6B). Although there were no significant changes in other metabolites in glycolysis/gluconeogenesis pathway, metabolites in the upstream of this pathway, such as G6P [FC (*ob*/*ob*/WT) = 0.66, q value = 0.13], F6P [FC (*ob*/*ob*/WT) = 0.53, q value = 0.12], and F1,6P [FC (*ob*/*ob*/WT) = 0.79, q value = 0.14], slightly decreased in skeletal muscle of *ob*/*ob* mice, unlike in liver. Gapdh, Pgk1, and Pgam2 (phosphoglycerate mutase 2) were identified as decreased DEMEs in skeletal muscle of *ob*/*ob* mice (Figures 6B and S12B), and the gene expression of *Pgk1* and *Gapdh* was regulated at the transcript level by the decreased DRTFs such as *Rxra*, *Trp53*, and *Xbp1* (Figure 6B). Fbp2 was the only increased DEME in this pathway in skeletal muscle of *ob*/*ob* mice. The increase of Fbp2 in skeletal muscle may be involved in glycogenesis but not in gluconeogenesis because G6P is not converted to glucose because of lack of G6Pase expression (Gamberucci et al., 1996; Schaftingen and Gerin, 2002), unlike in liver. The expression of Gapdh and Pgk1 increased in liver of *ob*/*ob* mice, but decreased in skeletal muscle of *ob*/*ob* mice (Figures 6A and 6B), suggesting the possibility that the glycolysis pathway is potentially inhibited by decreased expression of some metabolic enzymes in skeletal muscle of *ob*/*ob* mice. Furthermore, succinate and PEP contributed inhibiting allosteric regulations in liver but activating regulations in skeletal muscle.

To construct the inter-organ metabolic cycles, we not only used the data from the two trans-omic subnetworks (Figures 6A and 6B) but also added data regarding the expression of genes encoding relevant transporters and DRTFs that regulate those genes from our transcriptomic analyses. We also used the proteomic data to identify relevant transporters in liver and skeletal muscle. For the glucose-alanine cycle, we added the *Asct1* and *Asct2* encoding amino acid transporters to both skeletal muscle and liver (Figure 6C) (Freidman et al., 2020; Kandasamy et al., 2018). Although both genes were increased in expression in both organs, the DRTFs responsible for their regulation differed and neither of those transporters were detected in our proteomic data. For the glucose-lactate cycle, we added the monocarboxylate transporter Slc16a1, a major transporter of lactate (Figure 6C) (Halestrap, 2013). Slc16a1 was a DEP that increased in liver but was unchanged in skeletal muscle.

The changes in alanine were not sufficient in liver or skeletal muscle for this metabolite to be a DEM (Figure 6C); although in skeletal muscle alanine was slightly decreased [FC (*ob*/*ob*/WT) = 0.59, q value = 0.10] and in liver alanine was slightly increased [FC (*ob*/*ob*/WT) = 1.21, q value = 0.20)]. Despite these limited changes in alanine in the organs, alanine decreased in the blood of *ob*/*ob* mice, suggesting either decreased release from skeletal muscle or increased uptake by liver. We found increased expression of the alanine transporter genes *Asct1* and *Asct2* in both liver and skeletal muscle, suggesting enhanced uptake in liver. The limited increase in alanine in liver was consistent with increased Gpt and Gpt2, which catalyzes at the conversion of alanine to pyruvate. In the glucose-lactate cycle, lactate was unchanged in skeletal muscle and the blood of *ob*/*ob* mice. However, in liver of *ob*/*ob* mice, lactate and its transporter, Slc16a1, increased (Figure 6C), suggesting the increased uptake of lactate from the blood to the liver. Ldha, which catalyzes lactate to pyruvate, also increased in liver of *ob*/*ob* mice (Figure 6C), consistent with the previous finding (Nesteruk et al., 2014). An increase of Gpt2 of the glucose-alanine cycle and Ldha of the glucose-lactate cycle in liver of *ob*/*ob* mice may increase the amount of pyruvate. Although we did not measure the amount of pyruvate, it has been reported that the hepatic pyruvate is actually increased in liver of overnight-fasted *ob*/*ob* mice (Shannon et al., 2021). Therefore, the increased Ldha and Gpt2 can contribute to the activation of gluconeogenesis through the conversion of lactate and alanine to pyruvate. Furthermore, expression of *Gpt2*, *Ldha*, and *Asct2* were upregulated by the DRTF *Pparg* (Figure 6C), suggesting that both glucose-alanine and glucose-lactate cycles in the liver are coordinately regulated at the transcriptional level. Collectively, these results suggested that increased hepatic glucose production occurs from both lactate and alanine in fasted *ob*/*ob* mice.

In addition to increased glucose in the blood in *ob*/*ob* mice, we found an increase in phosphorylation of As160 in skeletal muscle of *ob*/*ob* mice (Figures 2A and 6C). Given that As160 is a GTPase-activating protein (GAP) for Rab GTPases and its phosphorylation inhibits its GTPase activity, resulting in promoting Glut4 translocation into the plasma membrane via Rab GTPases, and consequently enhancing glucose transport (Huang and Czech, 2007), this result suggested the enhancement of glucose transport through Glut4 translocation. In addition, expression of *Slc2a4*/*Glut4* was slightly upregulated in *ob*/*ob* mice (Figure 6C). By contrast, we found reduced glycolysis in skeletal muscle as suggested by slightly decrease in glycolytic metabolites from G6P to PEP together with decreased glycolytic enzymes Gapdh, Pgk1, and Pgam2 and increased gluconeogenesis in liver as suggested by increased Gpt, Gpt2, and Ldha (Figures 6B and 6C). The two facts that increased blood glucose and phosphorylation of As160 and that decreased downstream glycolytic metabolites (G6P to PEP) are contradictory, implying an existence of dysregulation of glucose transport in skeletal muscle. Consistently, it has been shown that glucose uptake is reduced in skeletal muscle of *ob*/*ob* mice (Ohshima et al., 1984). These results suggest the possibility that Glut4 translocation to plasma membrane, which is downstream of As160, is impaired in *ob*/*ob* mice, leading to the opposite changes of blood glucose and glycolytic metabolites in skeletal muscle in *ob*/*ob* mice. Taken together, blood glucose and blood alanine showed the similar changes to those in the releasing organs (the liver for glucose, skeletal muscle for alanine and lactate) and the opposite changes to glucose, alanine, and lactate in the uptaking organs (skeletal muscle for glucose, the liver for alanine and lactate). This result suggests that abnormal changes of blood glucose, blood alanine, and blood lactate resulted from the imbalance of metabolic pathways in releasing and uptaking organs.

Thus, the trans-omic analysis of inter-organ metabolic cycles provided the potential mechanisms of obesity-associated dysfunction in cycles between liver and skeletal muscle in glucose-alanine and glucose-lactate and its potential dysregulation.

### Potential mechanisms of dysfunction of inter-organ metabolic cycles of ketone body and their dysregulation in the trans-omic network in liver and skeletal muscle of *ob*/*ob* mice

We investigated potential mechanisms of dysfunction of ketone body cycle in liver and skeletal muscle. We extracted the trans-omic subnetwork of fatty acid degradation pathway from the trans-omic networks in the liver and skeletal muscle according to information of “fatty acid degradation” (mmu00071) and “glycerolipid metabolism” (mmu00561) in the KEGG database (Kanehisa et al., 2017). TAG and DAG, which are intermediate lipids in the fatty acid degradation pathway, increased in the liver of *ob*/*ob* mice (Figure 7A), consistent with the previous findings (Yu et al., 2004; Perfield et al., 2013). Nine enzymes were increased DEMEs in the liver of *ob*/*ob* mice, whereas 4 enzymes were the decreased DEMEs. Among the increased DEMEs, Acsl5, Acadm, Ecsh1, Hadh, Hsd17b4, and Acaa1b showed the increased expression at the transcript level by the increased DRTFs, *Pparg*, *Srebf1*, *Esr1*, and *Klf4* (Figure 7A). Taken together, these results imply that fatty acid degradation pathway is potentially activated at enzyme level in the liver of *ob*/*ob* mice. In addition, there was some allosteric regulations by the DEMs such as ATP and GSH in the liver of *ob*/*ob* mice (3 activating and 4 inhibiting regulations) (Figure S12C). In skeletal muscle of *ob*/*ob* mice, TAG was also identified as the increased DEM (Figure 7B), consistent with the previous finding (Mastrocola et al., 2015). All eight DEMEs increased in skeletal muscle of *ob*/*ob* mice (Figures 7B and S12D), and of these increased DEMEs, Cpt2, Acadm, Acadl, Acadvl, and Ecsh1 have been previously reported to increase (Schönke et al., 2018). Expression of Hadhb and Acaa2 was regulated at the transcript level by the increased DRTFs such as *Nfatc2* and *Egr1* (Figure 7B). Acadm and Echs1 were identified as the increased DEMEs common both in the liver and skeletal muscle (Figures 7A and 7B). These results suggest that the fatty acid degradation pathway is potentially activated at enzyme expression level both in liver and skeletal muscle.

To explore ketone body cycle in *ob*/*ob* mice, we added the genes for the monocarboxylate transporters *Slc16a7* to skeletal muscle and *Slc16a6* to liver (Figure 7C) (Newman and Verdin, 2014). *Slc16a7* expression decreased in skeletal muscle, suggesting reduced capacity for ketone body transport. In contrast, *Slc16a6* expression increased in liver, consistent with the previous report using overnight-fasted *db*/*db* mice (Kim et al., 2016), suggesting an increased capacity for ketone body transport. We found that the DEMEs responsible for fatty acid degradation (Figure 7A) and ketone bodies (acetoacetate and 3-OH butyrate) production, such as Hmgcl (3-hydroxy-3-methylglutaryl-CoA lyase), Bdh1 (D-beta-hydroxybutyrate dehydrogenase), and a rate-limiting enzyme of ketone body synthesis Hmgcs2 (3-hydroxy-3-methylglutaryl-CoA synthase 2), increased in liver of *ob*/*ob* mice (Figure 7C). This result suggests that the synthesis of 3-OH butyrate is potentially activated in liver of *ob*/*ob* mice, although the metabolites for ketone body production, such as acetyl-CoA and 3-OH butyrate, did not significantly change. Further study is necessary to address whether the synthesis rate of 3-OH butyrate is activated in liver of *ob*/*ob* mice. Skeletal muscle exhibited reduced capacity for ketone body uptake (decreased *Slc16a7* expression) and ketone body metabolism as suggested by decreased Bdh1 (Figure 7C). Despite this increased capacity to produce and release the ketone body 3-OH butyrate by the liver and the decreased use of this ketone body by skeletal muscle from *ob*/*ob* mice, blood concentrations of 3-OH butyrate were unchanged from WT mice.

## Discussion

We measured multiple omic data—transcriptomic, proteomic, and metabolomic—in liver and skeletal muscle of WT and *ob*/*ob* mice during a fasting state and identified differentially expressed molecules that significantly changed in *ob*/*ob* mice relative to WT in each omic layer and connected these layers with differential regulations. From this large set of data, we extracted differentially expressed molecules and differential regulations that are involved in metabolic reactions and constructed a trans-omic network for liver and a network for skeletal muscle of the differentially regulated metabolic reactions. By integrating the trans-omic networks in liver and skeletal muscle with metabolomic data from blood, we explored the potential mechanisms of dysfunction of three inter-organ metabolic cycles: glucose-alanine, glucose-lactate, and ketone bodies.

We provided the potential mechanisms of obesity-associated dysfunctions and dysregulations of inter-organ metabolic cycles of glucose-alanine and glucose-lactate: (1) activation of the gluconeogenesis pathway both from alanine and lactate by increased enzyme expression in the liver and (2) impaired glycolysis by decreased enzyme expression in skeletal muscle. In the activation of gluconeogenesis pathway in liver of *ob*/*ob* mice, expression of *Gpt2* and *Ldha*, encoding enzymes that catalyze alanine and lactate to pyruvate, respectively, and of *Asct2*, encoding an alanine transporter, were upregulated by the common DRTF, *Pparg*, suggesting a mechanism for coordinated activation of these dysregulated processes in liver. Taken together, the data suggested that dysregulations of the glucose-alanine and glucose-lactate cycles involve activation of gluconeogenesis in liver and inhibition of glycolysis in skeletal muscle, which may contribute to hyperglycemia of *ob*/*ob* mice. However, further studies are required to elucidate the causal relationship between obesity-associated changes of the activation status of pathways and dysregulation of the inter-organ metabolic cycles. Moreover, we found that blood glucose and phosphorylation of As160 in skeletal muscle increased, whereas downstream glycolytic metabolites (G6P to PEP) decreased, suggesting that Glut4 translocation to plasma membrane, which is downstream of As160, may be impaired in *ob*/*ob* mice. This may be the key mechanism of insulin resistance in skeletal muscle of *ob*/*ob* mice.

We provided the potential mechanisms of dysregulations of the inter-organ metabolic cycle of ketone body; both the production of a ketone body, 3-OH butyrate, in liver and its release from the liver into the blood increased in *ob*/*ob* mice, suggesting that excess lipids are converted into this ketone body in the liver of *ob*/*ob* mice. In addition, the expression of the gene encoding the 3-OH butyrate transporter in skeletal muscle of *ob*/*ob* mice decreased. However, the amount of 3-OH butyrate in the blood was similar between WT and *ob*/*ob* mice, suggesting that organs other than skeletal muscle take up this ketone body, preventing its accumulation in the blood despite increased production and release by the liver. In skeletal muscle of *ob*/*ob* mice, the expression of ketone body transporter and metabolic enzyme and the amount of 3-OH butyrate decreased, despite activation of enzymes involved in fatty acid degradation in skeletal muscle. These results suggest that fatty acids instead of ketone bodies were metabolized to produce acetyl-CoA in skeletal muscle. In skeletal muscle from *ob/ob* mice, 5 of the 8 increased DEMEs involved in fatty acid degradation were not upregulated at the transcript level (see Figure 7B), suggesting that the increased abundance of these enzymes may be controlled by the modulation of protein turnover.

The increased DRTF *Klf4* regulated the expression of the increased DEGs encoding metabolic enzymes involved in fatty acid degradation (*Ascl5*, *Acadm*, *Echs1*, and *Acaa1b*) and of *Hmgcl* (encoding HMG-CoA lyase involved in ketone body synthesis) in liver of *ob*/*ob* mice. Klf4 is a crucial regulator of mitochondrial homeostasis in cardiac muscle (Liao et al., 2015), but its role in organismal or liver metabolism is still largely unknown. Klf4 may be an important regulator contributing to ketone body production in liver of *ob*/*ob* mice.

In the trans-omic network in the liver, the activating regulation by DEMEs and the inhibiting allosteric regulation by the DEMs counteract each other, implying that the liver may adapt to obesity-induced changes in metabolites with compensatory increases in metabolic enzymes induced by increased gene expression. Expression of many of the increased DEMEs were also regulated at the gene expression level by *Pparg*, *Srebf1*, *Klf4*, and *Esr1*. Both Pparg and Srebp1 are key regulators of hepatic lipid metabolism, and those TFs are highly expressed in liver of obese individuals (Bedoucha et al., 2001; Shimomura et al., 1999; Jeon and Osborne, 2012). Consistent with the earlier observations, we also identified increased expression of *Pparg* and *Srebf1* in liver of *ob*/*ob* mice and increased activation of lipid metabolic pathways regulated by their target genes. *Pparg* and *Srebf1* also activated genes involved in carbohydrate metabolism, including those in the glycogen, TCA cycle, and glycolysis and gluconeogenesis pathways, in the *ob*/*ob* mice, suggesting that these DRTFs coordinate altered lipid and carbohydrate metabolism in obesity. These results provide a potential transcriptional mechanism for the increased expression of genes encoding metabolic enzymes involved in carbohydrate metabolism that is associated with insulin resistance in obesity (Guo, 2014; Roden and Shulman, 2019).

Changes in DEMs in liver and skeletal muscle of *ob*/*ob* mice, as well as in the blood, suggested higher oxidative stress in the *ob*/*ob* mice. In skeletal muscle, we identified anserine and carnosine, which are dipeptides composed of beta-alanine and histidine, as decreased DEMs in skeletal muscle of *ob*/*ob* mice. Anserine and carnosine are antioxidant factors involved in the degradation of lipid peroxide and are abundant in skeletal muscle (Kohen et al., 1988; Boldyrev et al., 2013). The decrease in these metabolites in *ob*/*ob* mice suggested that lipid peroxide may accumulate in skeletal muscle cells of *ob*/*ob* mice. In addition, both glutathione (GSH) and GSSG increased in the liver, and GSSG increased in the blood of *ob*/*ob* mice, which may reflect the oxidative stress associated with higher energy production in *ob*/*ob* mice (Tangvarasittichai, 2015).

To comprehensively elucidate potential regulatory mechanisms of systemic metabolic homeostasis, inter-organ metabolic cycles for additional key metabolic organs should be evaluated. Adipose tissue is an obvious choice. We analyzed the trans-omic network of insulin signaling in differentiated 3T3 adipocytes (Krycer et al., 2017; Quek et al., 2020; Ohno et al., 2020). We intend to apply our trans-omic approach to investigating inter-organ metabolic cycles between adipose tissue and other organs *in vivo* in the future.

Several multi-omic studies, including our previous trans-omic works, examined the effects of obesity on metabolism in human or animal models (Kokaji et al., 2020; Parks et al., 2015; Soltis et al., 2017; Williams et al., 2016). Soltis et al. performed epigenomic, transcriptomic, proteomic, and metabolomic analyses of liver from mice fed a normal or high-fat diet. Some of the differentially expressed molecules associated with high-fat diet in their study were consistent with our results from *ob*/*ob* mice in previous study (Kokaji et al., 2020) and the present study. In the amount of metabolite, GSH and lactate increased, and Gly decreased in the liver of both obese mice (Kokaji et al., 2020; Soltis et al., 2017). In the expression of genes, the increased DEGs in both obese mice were enriched in glycolysis/gluconeogenesis and steroid synthesis, whereas the decreased DEGs in both obese mice were enriched in 4 amino acid metabolic pathways such as Ala, Asp, and Glu metabolism and Gly, Ser, and Thr metabolism (Kokaji et al., 2020; Soltis et al., 2017). Besides, we compared our DETFs with TFs in liver of high-fat diet-induced obese mice (Soltis et al., 2017) (Figures 2F and S13). We found that more than half of the DETF were similarly regulated in the high-fat diet-induced mice (3 out of 4 increased DETFs and 3 out of 6 decreased DETFs). In particular, the increases of *Pparg*, *Srebf1*, and *Esr1* and the decreases of *Zfx*, *Gtf2ird1*, and *Trim28* consistently occurred both in their high-fat-diet-fed mice and in our *ob*/*ob* mice fed with normal diet. Because these changes occurred in response to both high-fat diet and the absence of leptin (*ob*/*ob*), this result indicates that these DETFs are associated with obesity, not with leptin deficiency nor with the content of diet. The present study extends the previous multi-omic and trans-omic studies by including skeletal muscle and evaluating inter-organ metabolic cycles, thereby providing distinct insights into dysregulated metabolism in obesity.

The analysis of trans-omic networks for differentially regulated metabolic reactions can be applied to elucidate trans-omic network using pathological models other than obesity and potentially contribute to the discovery of novel therapeutic targets. In the future, assembling larger-scale trans-omic networks for differentially regulated metabolic reactions between healthy and obese individuals by adding to multi-omic datasets will uncover the whole picture of the effects of obesity on systemic metabolic homeostasis.

### Limitations of the study

The potential mechanisms of the dysregulation of the inter-organ metabolic cycles in this study may not reflect direct causal relationship and should be validated by further experiments in the future. Limitations of our study include that we evaluated only liver and skeletal muscle and only at a single time point of fasting. More detailed investigation of the effects of obesity on metabolite exchange across each organ will require the acquisition of metabolite time series data using isotope tracers (Fernández-García et al., 2020), along with acquisition of transcriptomic and proteomic data at multiple time points. The sample size of lipidome (n = 3) is relatively small compared with other omic analyses, and the statistical power is low for lipidomic analysis. For this reason, we did not include the lipidomic data in the trans-omic networks (Figure 4). In addition, we focused on metabolic reactions. However, perturbations in liver and skeletal muscle of *ob*/*ob* mice are not limited to metabolism. These mice also exhibit increased chronic inflammation or disrupted organ architecture (Cohen et al., 2011). We need to analyze other perturbed cellular functions than metabolism for comprehensive understanding of systemic changes in *ob*/*ob* mice. For example, skeletal muscle is primarily a contractile organ. Thus, these cells may adapt to obesity through changes in non-metabolic cellular functions, such as muscle contraction. Indeed, we found pathways involved in contractile function, such as the regulation of actin cytoskeleton, from pathway enrichment analysis in DEGs in skeletal muscle (Table S2) and identified increased DEPs associated with muscle contraction, such as Myh2 and Myh7 (see Figure 2D). There are two types of regulation of TFs: regulation by expression change and regulation by posttranslational modifications including, phosphorylation (differentially phosphorylated TFs (DPTFs)). In this study, we identified DETFs using RNA-seq and included only pFoxo1 as a DPTF based on the literature. In the future, the phosphoproteomic measurement will allow us to comprehensively identify DPTFs according to our methods (Yugi et al., 2014; Kawata et al., 2019). Identification of both DETFs and DPTFs will enable us to examine how much of gene expression is controlled by DETFs and DPTFs in *ob*/*ob* mice. Thus, large-scale measurements of phosphorylation levels of metabolic molecules by the phosphoproteomics are required (Yugi et al., 2014; Kawata et al., 2019; Humphrey et al., 2013). We also only evaluated TFs as upstream regulators of the gene expression network. Yet, considerations of epigenomic controls, such as histone modifications and DNA methylation, will provide us with further insight into the organ-specific traits of gene expression regulatory network (Ahrens et al., 2013; Kirchner et al., 2016). RNA-mediated regulation, such as through miRNA or lncRNA, is also likely and will need to be added to the regulatory network to enable comprehensive analyses.

### Resource availability

#### Lead contact

Further information and requests for resources and reagents should be directed to and will be fulfilled by the lead contact, Shinya Kuroda (skuroda@bs.s.u-tokyo.ac.jp).

#### Materials availability

This study did not generate any new material.

#### Data and code availability

The accession number for the sequencing data of the liver and skeleltal muscle reported in this paper is the DNA DataBank of Japan Sequence Read Archive (DRA) (www.ddbj.nig.ac.jp/): DRA008416, DRA010972. The accession number for the data of proteome analysis reported in this paper is the ProteomeXchange Consortium (http://proteomecentral.proteomexchange.org) via the JPOST partner repository: PXD020656. All other data are available with the published article. The code used for the analysis in this paper is available upon request.

## Methods

All methods can be found in the accompanying transparent methods supplemental file.

## Supplemental information

Document S1. Transparent methods and Figures S1–S13

Table S1. Western blotting data in the liver and skeletal muscle, related to figure 2

Table S2. Transcriptomic analysis in the liver and skeletal muscle, related to figure 2

Table S3. Proteomic analysis in the liver and skeletal muscle, related to figure 2

Table S4. Metabolomic analysis in the liver and skeletal muscle, related to figure 2

Table S5. Lipidomic analysis in the liver and skeletal muscle, related to figure 2

Table S6. Transcription factor enrichment analysis in the liver and skeletal muscle, related to figure 2

Table S7. KEGG pathway enrichment analysis on genes encoding the differentially expressed proteins showing the differences at the transcript level in the liver of ob/ob mice, related to figure 3

Table S8. Metabolomic analysis in the blood, related to figure 5

## Footnotes

Supplemental information can be found online at https://doi.org/10.1016/j.isci.2021.102217.
