# Supplementary material for: Trans-omic analysis reveals obesity-associated dysregulation of inter-organ metabolic cycles between the liver and skeletal muscle
Source: iScience. 2021 Feb 20;24(3):102217. doi: 10.1016/j.isci.2021.102217 (PMC7961104; doi:10.1016/j.isci.2021.102217)
Supplement: Document S1. Transparent methods and Figures S1–S13 [file mmc1.pdf]

**iScience, Volume *24***

## Supplemental information

# Trans-omic analysis reveals obesity-associated dysregulation of inter-organ metabolic cycles between the liver and skeletal muscle

**Riku Egami, Toshiya Kokaji, Atsushi Hatano, Katsuyuki Yugi, Miki Eto, Keigo Morita, Satoshi Ohno, Masashi Fujii, Ken-ichi Hironaka, Saori Uematsu, Akira Terakawa, Yunfan Bai, Yifei Pan, Takaho Tsuchiya, Haruka Ozaki, Hiroshi Inoue, Shinsuke Uda, Hiroyuki Kubota, Yutaka Suzuki, Masaki Matsumoto, Keiichi I. Nakayama, Akiyoshi Hirayama, Tomoyoshi Soga, and Shinya Kuroda**

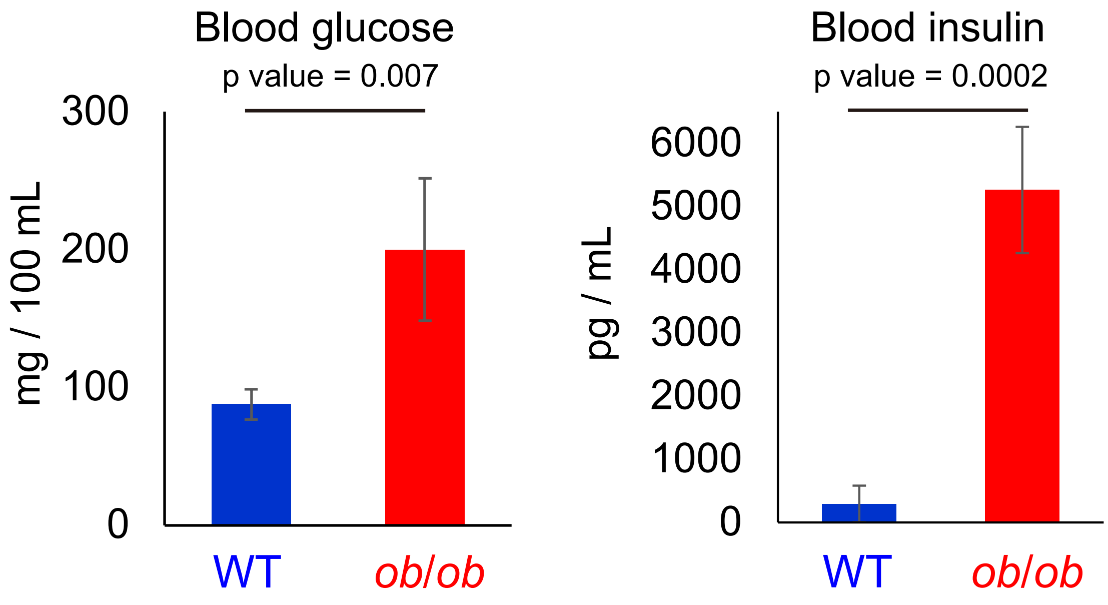


**Figure S1. Glucose and Insulin Concentration in the Blood of the Wild-type (WT) and *ob*/*ob* Mice. Related to Figure 1.**
The concentrations of glucose (Left) and insulin (Right) in the blood of 16 hours-fasted WT and *ob*/*ob* mice (Kokaji et al., 2020). Blue and red bars represent concentrations in WT and *ob*/*ob* mice, respectively. The means and standard error of the means (SEMs) of 5 mice are shown.

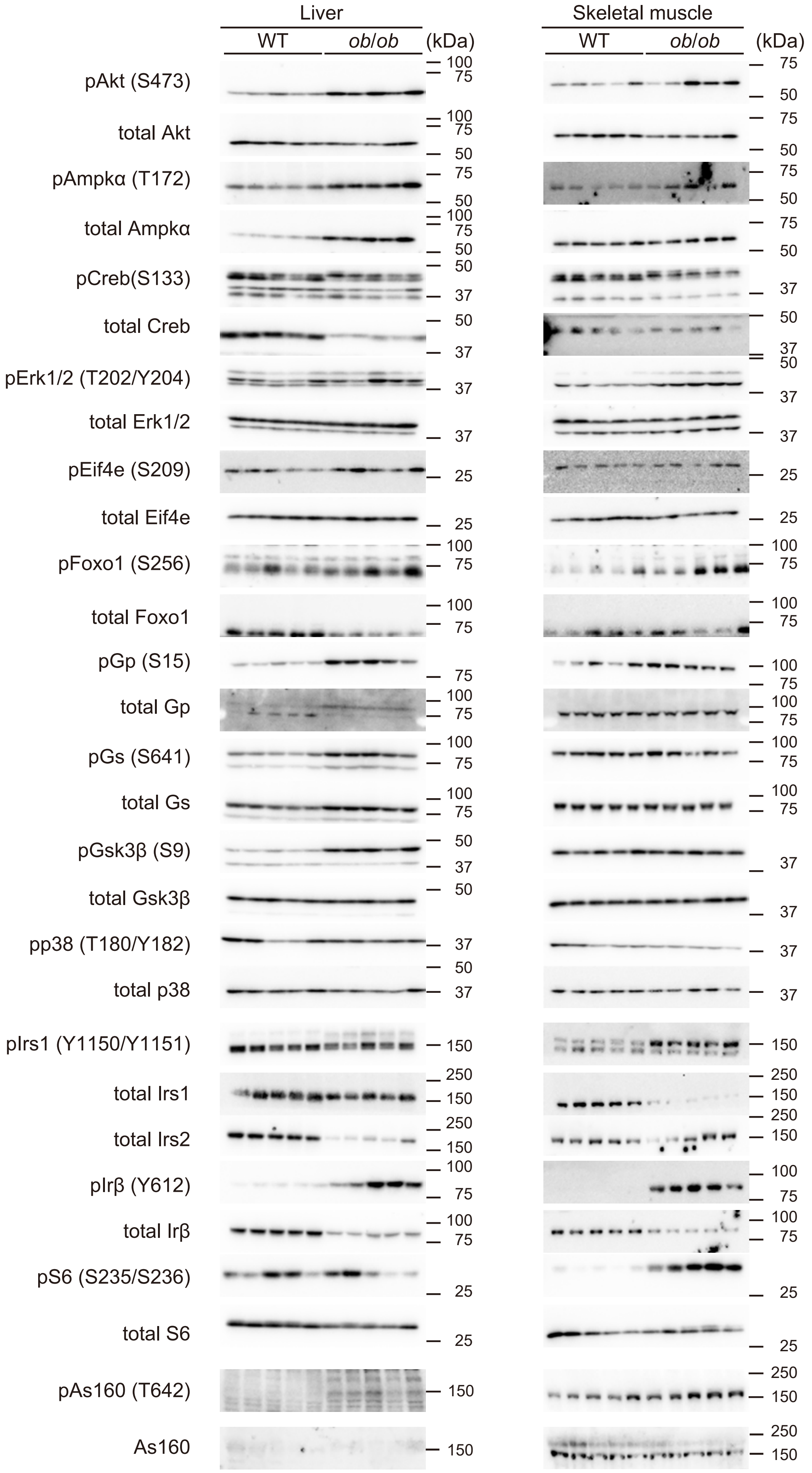


**Figure S2. Western Blotting in Liver and Skeletal Muscle. Related to Figure 2.**

The amount and phosphorylation of the indicated molecules in the liver and skeletal muscles of WT mice and *ob*/*ob* mice. All western blot data of 5 mice are shown. The unabbreviated name of the molecules can be found in Table S1.

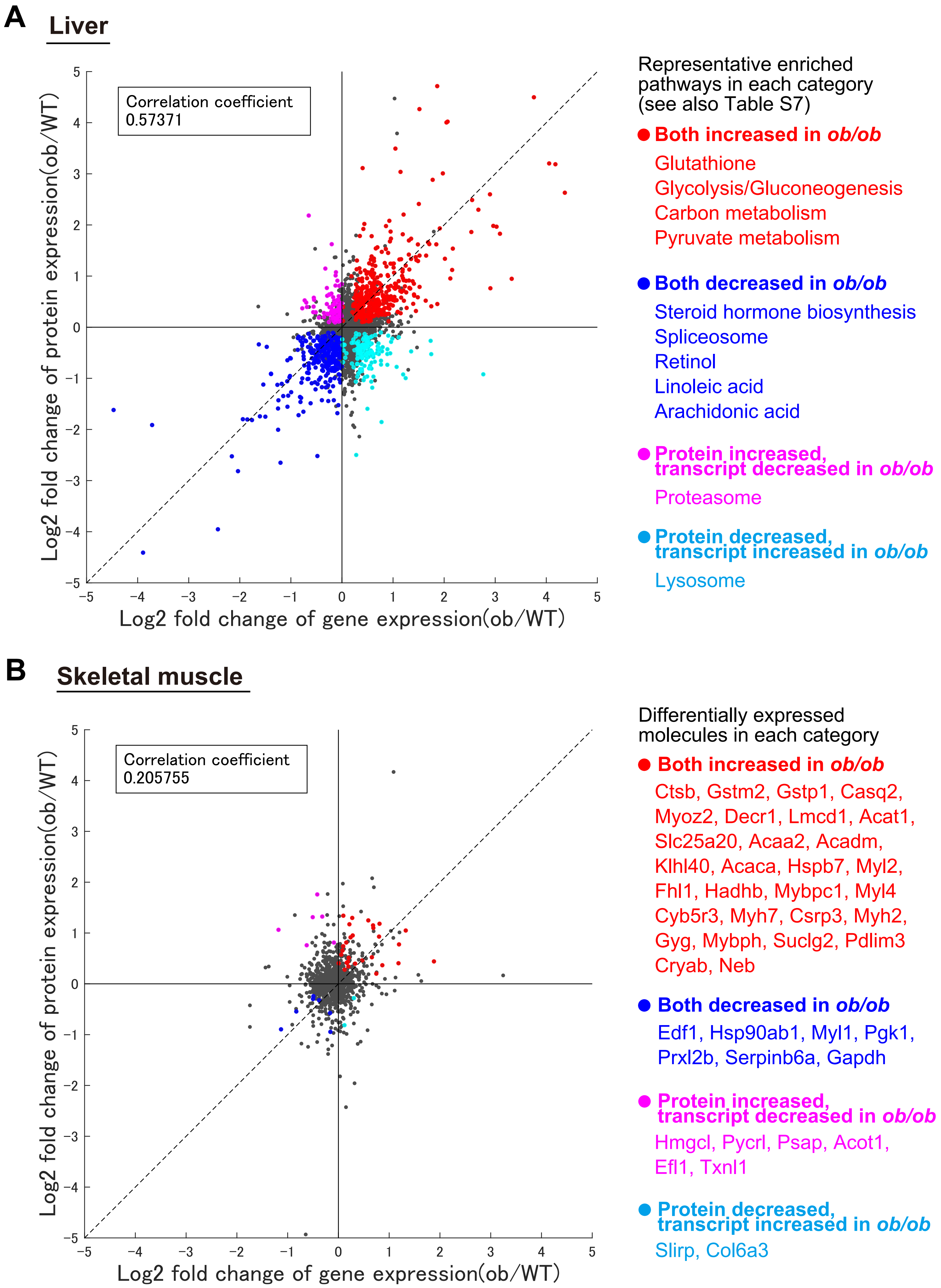
A
Liver
Correlation coefficient
0.57371
Log2 fold change of protein expression(ob/WT)
Log2 fold change of gene expression(ob/WT)
5
4
3
2
1
0
−1
−2
−3
−4
−5
Representative enriched pathways in each category (see also Table S7)
Both increased in ob/ob
Glutathione
Glycolysis/Gluconeogenesis
Carbon metabolism
Pyruvate metabolism
Both decreased in ob/ob
Steroid hormone biosynthesis
Spliceosome
Retinol
Linoleic acid
Arachidonic acid
Protein increased, transcript decreased in ob/ob
Proteasome
Protein decreased, transcript increased in ob/ob
Lysosome
B
Skeletal muscle
Correlation coefficient
0.205755
Log2 fold change of protein expression(ob/WT)
Log2 fold change of gene expression(ob/WT)
Differentially expressed molecules in each category
Both increased in ob/ob
Ctsb, Gstm2, Gstp1, Casq2, Myoz2, Decr1, Lmcd1, Acat1, Slc25a20, Acaa2, Acadm, Klhl40, Acaca, Hspb7, Myl2, Fhl1, Hadhb, Mybpc1, Myl4 Cyb5r3, Myh7, Csrp3, Myh2, Gyg, Mybph, Suclg2, Pdlim3 Cryab, Neb
Both decreased in ob/ob
Edf1, Hsp90ab1, Myl1, Pgk1, Prxl2b, Serpinb6a, Gapdh
Protein increased, transcript decreased in ob/ob
Hmgcl, Pycrl, Psap, Acot1, Efl1, Txnl1
Protein decreased, transcript increased in ob/ob
Slirp, Col6a3

**Figure S3. Integrative Analysis of Proteome and Transcriptome in Liver and Skeletal Muscle. Related to Figure 3.**
(A, B) The $\log_2$ fold changes of measured molecules in the liver (A) and skeletal muscle (B) between WT and *ob*/*ob* mice. Each dot on a scatter plot was colored according to the variation of each molecule: red dots, increased molecules in *ob*/*ob* mice both at transcript and protein level; blue dots, decreased molecules in *ob*/*ob* mice both at transcript and protein level; magenta dots, molecules with increased at protein level and decreased at transcript level in *ob*/*ob* mice; cyan dots, molecules with decreased at protein level and increased at transcript level in *ob*/*ob* mice. In analysis of the liver, several representative pathways found by KEGG pathway enrichment analysis are displayed on the right of Figure S3A (see also Table S8). In skeletal muscle, names of gene are displayed on the right of Figure S3B.

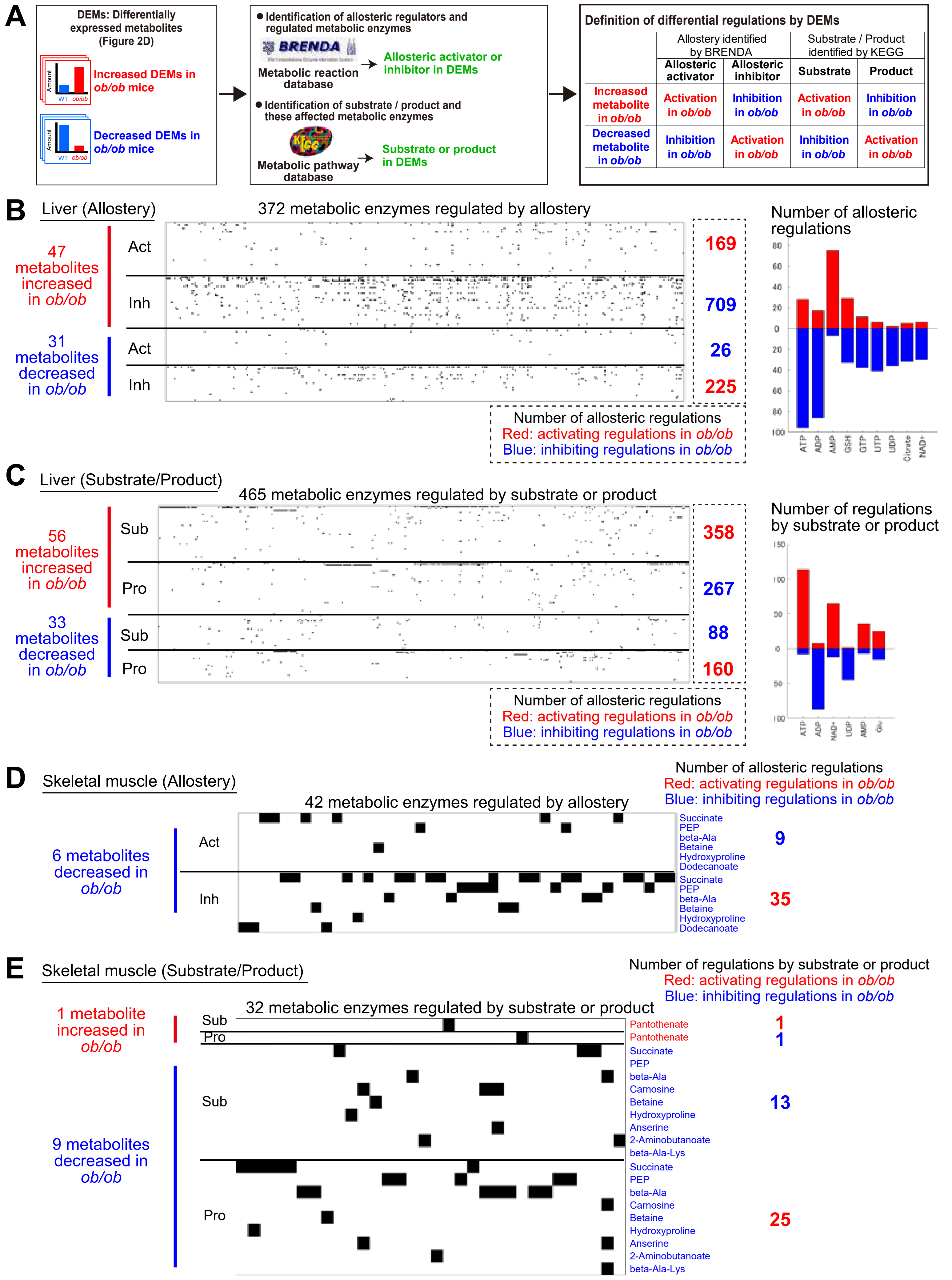
A
DEMs: Differentially expressed metabolites (Figure 2D)
Increased DEMs in ob/ob mice
Decreased DEMs in ob/ob mice
Amount
WT ob/ob
Identification of allosteric regulators and regulated metabolic enzymes
BRENDA
Metabolic reaction database
Allosteric activator or inhibitor in DEMs
Identification of substrate / product and these affected metabolic enzymes
Metabolic pathway database
Substrate or product in DEMs
Definition of differential regulations by DEMs
Allostery identified by BRENDA
Substrate / Product identified by KEGG
Allosteric activator
Allosteric inhibitor
Substrate
Product
Increased metabolite in ob/ob
Activation in ob/ob
Inhibition in ob/ob
Activation in ob/ob
Inhibition in ob/ob
Decreased metabolite in ob/ob
Inhibition in ob/ob
Activation in ob/ob
Inhibition in ob/ob
Activation in ob/ob
B
Liver (Allostery)
372 metabolic enzymes regulated by allostery
47 metabolites increased in ob/ob
31 metabolites decreased in ob/ob
Act
Inh
Act
Inh
169
709
26
225
Number of allosteric regulations
Red: activating regulations in ob/ob
Blue: inhibiting regulations in ob/ob
Number of allosteric regulations
ATP
ADP
AMP
GSH
GTP
UTP
UDP
Citrate
NAD+
C
Liver (Substrate/Product)
465 metabolic enzymes regulated by substrate or product
56 metabolites increased in ob/ob
33 metabolites decreased in ob/ob
Sub
Pro
Sub
Pro
358
267
88
160
Number of allosteric regulations
Red: activating regulations in ob/ob
Blue: inhibiting regulations in ob/ob
Number of regulations by substrate or product
ATP
ADP
NAD+
UDP
AMP
Glu
D
Skeletal muscle (Allostery)
42 metabolic enzymes regulated by allostery
6 metabolites decreased in ob/ob
Act
Inh
Number of allosteric regulations
Red: activating regulations in ob/ob
Blue: inhibiting regulations in ob/ob
Succinate
PEP
beta-Ala
Betaine
Hydroxyproline
Dodecanoate
9
Succinate
PEP
beta-Ala
Betaine
Hydroxyproline
Dodecanoate
35
E
Skeletal muscle (Substrate/Product)
32 metabolic enzymes regulated by substrate or product
1 metabolite increased in ob/ob
9 metabolites decreased in ob/ob
Sub
Pro
Sub
Pro
Number of regulations by substrate or product
Red: activating regulations in ob/ob
Blue: inhibiting regulations in ob/ob
Pantothenate
Pantothenate
1
1
Succinate
PEP
beta-Ala
Carnosine
Betaine
Hydroxyproline
Anserine
2-Aminobutanoate
beta-Ala-Lys
13
Succinate
PEP
beta-Ala
Carnosine
Betaine
Hydroxyproline
Anserine
2-Aminobutanoate
beta-Ala-Lys
25

**Figure S4. Identification of Allosteric Regulations and Regulation by Substrate or Product in Liver and Skeletal Muscle. Related to Figure 3.**

(A) The procedure for identification of differential regulations for metabolic reactions by differentially expressed metabolites (DEMs). We identified allosteric regulations by the allosteric regulators among the DEMs to metabolic reactions using BRENDA (Jeske et al., 2019) and the substrates and products among the DEMs that can affect metabolic reactions using KEGG (Kanehisa et al., 2017). The differential regulations by the DEMs are classified into either activating or inhibiting regulations in *ob*/*ob* mice, which are determined by the combination of the direction of changes of the DEMs (increase or decrease) and of direction of regulation (activator or inhibitor).

(B) Allosteric regulations in the liver to metabolic enzymes by allosteric regulators (Left). The row labels represent each allosteric regulator that increased or decreased in *ob*/*ob* mice and are classified into either activator or inhibitor. The column labels show metabolic enzymes regulated by allostery. A black dot is provided at row *i* column *j* if a metabolic enzyme of column *j* is regulated by an allosteric regulator of row *i*. The number of allosteric regulations to metabolic reaction by the indicated allosteric regulator (Right). Only the regulations whose number is 30 or more are shown in bar plot.

(C) Regulations by substrate or product in the liver to metabolic enzymes by differentially expressed substrate or product (Left). The row labels represent each substrate or product that increased or decreased in *ob*/*ob* mice. The column labels show metabolic enzymes affected by differentially expressed substrate or product. A black dot is provided at row *i* column *j* if a metabolite of row *i* is a substrate or a product of a metabolic enzyme of column *j*. The number of regulations by the indicated substrate or product (Right). Only the regulations whose number is 30 or more are shown in bar plot.

(D) Allosteric regulations in skeletal muscle to metabolic enzymes by allosteric regulators.

(E) Regulations by substrate or product in skeletal muscle to metabolic enzymes by differentially expressed substrate or product.

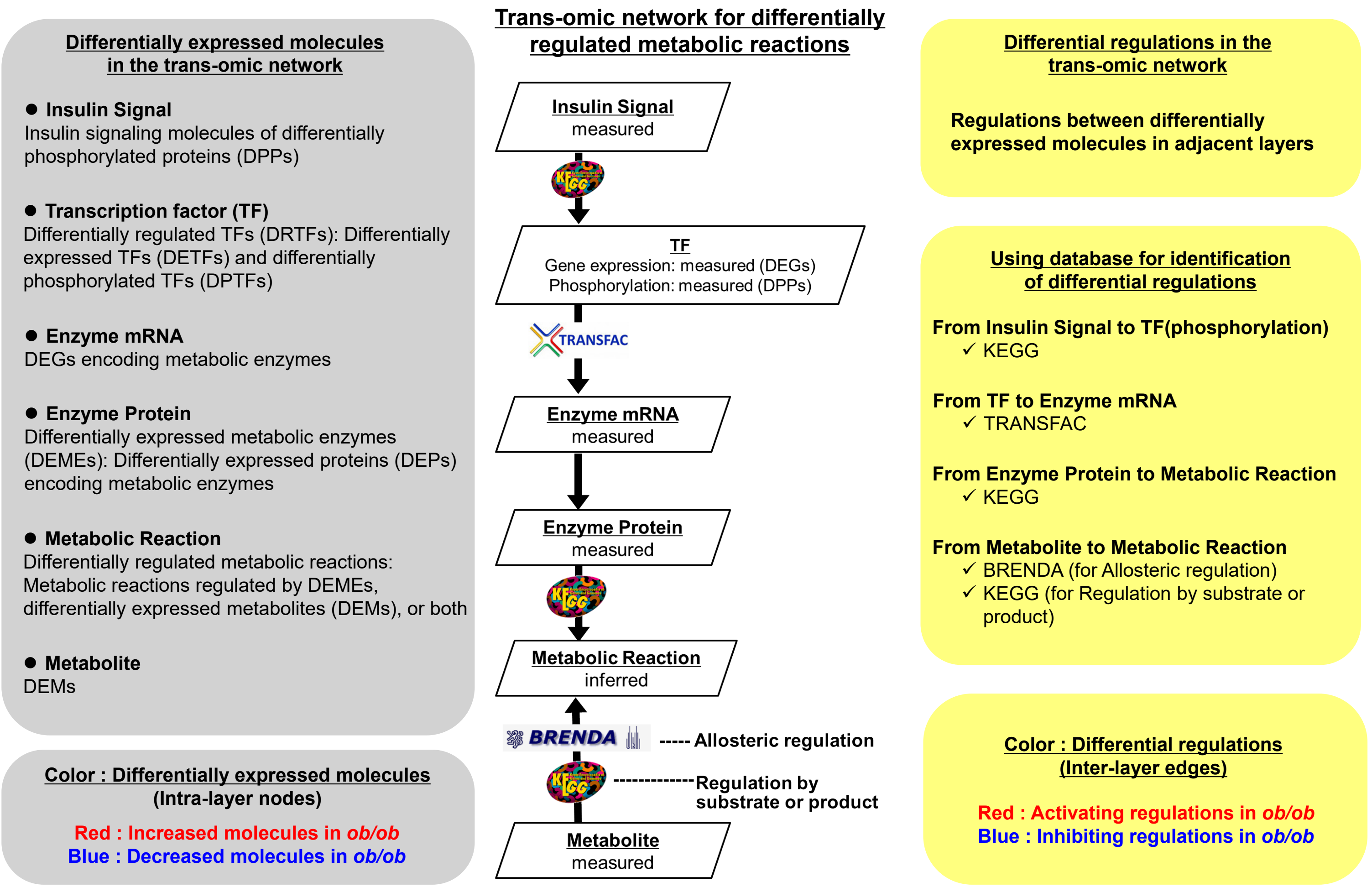


**Differential regulations in trans-omic network**

| Regulating differentially expressed molecules in trans-omic network | | | Regulated: TF(Phosphorylation) Increased | TF(Phosphorylation) Decreased | Enzyme mRNA Increased | Enzyme mRNA Decreased | Enzyme Protein Increased | Enzyme Protein Decreased | Metabolic Reaction |
|---|---|---|---|---|---|---|---|---|---|
| | Insulin Signal (Activator) | Increased | Activating | - | | | | | |
| | | Decreased | - | Inhibiting | | | | | |
| | Insulin Signal (Inhibitor) | Increased | - | Inhibiting | | | | | |
| | | Decreased | Activating | - | | | | | |
| | TF | Activated | | | Activating | - | | | |
| | | Inhibited | | | - | Inhibiting | | | |
| | Enzyme mRNA | Increased | | | | | Activating | - | |
| | | Decreased | | | | | - | Inhibiting | |
| | Enzyme Protein | Increased | | | | | | | Activating |
| | | Decreased | | | | | | | Inhibiting |
| | Metabolite (Allosteric activator) | Increased | | | | | | | Activating |
| | | Decreased | | | | | | | Inhibiting |
| | Metabolite (Allosteric inhibitor) | Increased | | | | | | | Inhibiting |
| | | Decreased | | | | | | | Activating |
| | Metabolite (Substrate) | Increased | | | | | | | Activating |
| | | Decreased | | | | | | | Inhibiting |
| | Metabolite (Product) | Increased | | | | | | | Inhibiting |
| | | Decreased | | | | | | | Activating |

**Figure S5. Contents of the Trans-Omic Network for Differentially Regulated Metabolic Reactions between WT and *ob*/*ob* Mice. Related to Figure 4.**

The layers of the Insulin Signal, Transcription Factor (TF), Enzyme mRNA, Enzyme Protein, and Metabolite correspond to the indicated differentially expressed molecules. The Metabolic Reaction layer represents differentially regulated metabolic reactions, which are defined as metabolic reactions directly regulated by the differentially expressed molecules in the Enzyme Protein layer and those in the Metabolite layer. The upward and downward arrows indicate the differential regulations between differentially expressed molecules in each omic layer. The databases used to identify the differential regulations are shown on the arrows. The left panel of the diagram showed the definitions of the differentially expressed molecules included in each layer (upper) and the color annotations according to the classification of the differentially expressed molecules (lower). The right panel of the diagram shows the definition of differential regulations (upper), the database used to identify differential regulations (middle), and the color annotations according to the classification of differential regulations (lower), respectively. The lower table represents the classification of the differential regulations in trans-omic network. The differential regulations are classified into either activating or inhibiting regulations in *ob*/*ob* mice, which are determined by the combination of the direction of changes of differentially expressed molecules (increased or decreased) and of direction of regulation (activator or inhibitor).

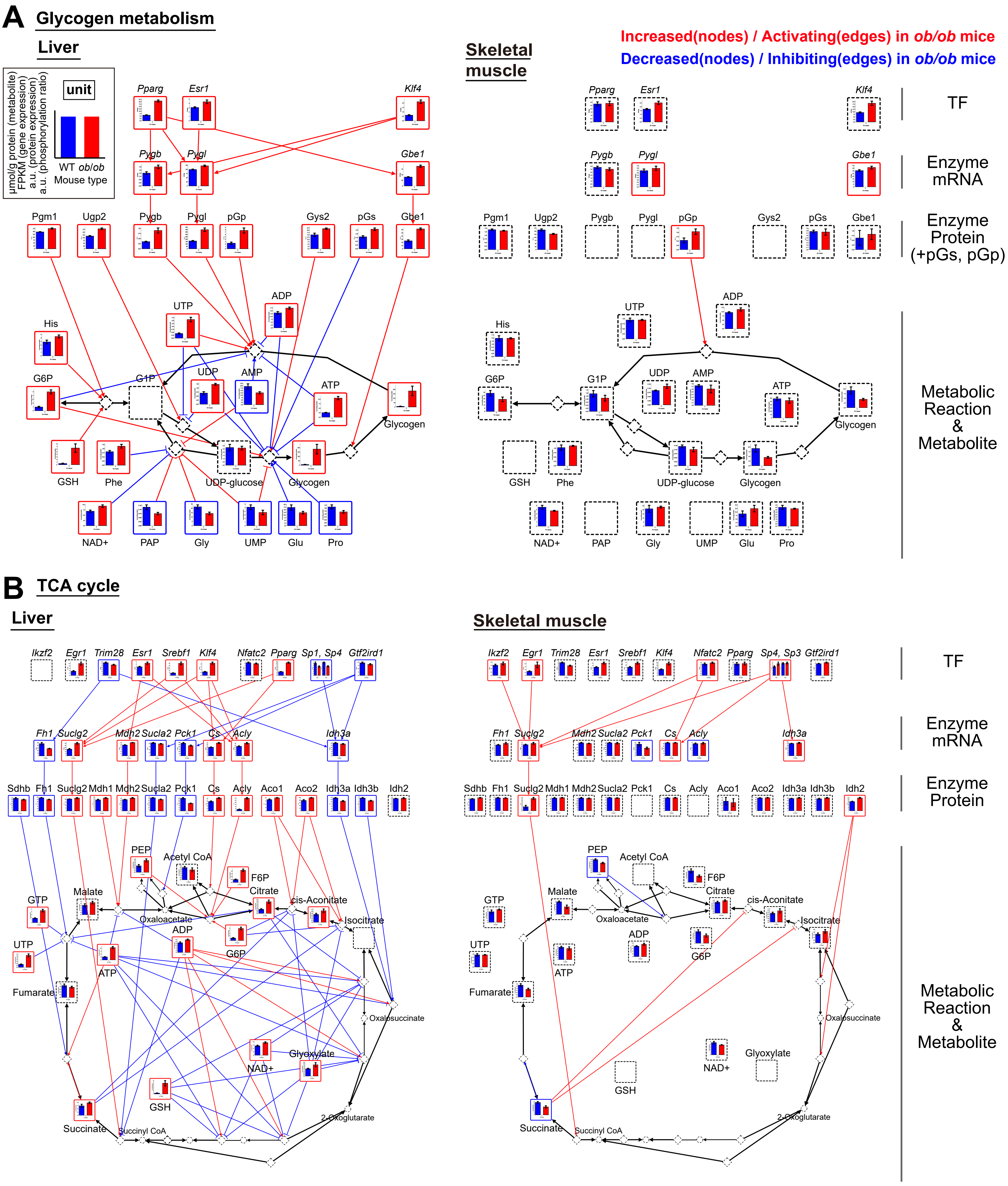
A Glycogen metabolism
Liver
unit
µmol/g protein (metabolite)
FPKM (gene expression)
a.u. (protein expression)
a.u. (phosphorylation ratio)
WT ob/ob
Mouse type
Skeletal muscle
Increased(nodes) / Activating(edges) in ob/ob mice
Decreased(nodes) / Inhibiting(edges) in ob/ob mice
TF
Enzyme mRNA
Enzyme Protein (+pGs, pGp)
Metabolic Reaction & Metabolite
Pparg
Esr1
Klf4
Pygb
Pygl
Gbe1
Pgm1
Ugp2
pGp
Gys2
pGs
ADP
UTP
His
G6P
G1P
UDP
AMP
ATP
Glycogen
GSH
Phe
UDP-glucose
NAD+
PAP
Gly
UMP
Glu
Pro
B TCA cycle
Liver
Skeletal muscle
TF
Enzyme mRNA
Enzyme Protein
Metabolic Reaction & Metabolite
Ikzf2
Egr1
Trim28
Esr1
Srebf1
Klf4
Nfatc2
Pparg
Sp1, Sp4
Gtf2ird1
Sp4, Sp3
Fh1
Suclg2
Mdh2
Sucla2
Pck1
Cs
Acly
Idh3a
Sdhb
Mdh1
Aco1
Aco2
Idh3b
Idh2
PEP
Acetyl CoA
F6P
Malate
Citrate
GTP
cis-Aconitate
Oxaloacetate
Isocitrate
ADP
UTP
G6P
ATP
Fumarate
Oxalosuccinate
NAD+
Glyoxylate
GSH
Succinate
Succinyl CoA
2-Oxoglutarate

**Figure S6. The Trans-Omic Network for Differentially Regulated Metabolic Reactions in Glycogen Metabolism and TCA cycle. Related to Figure 4.**

(A, B) The trans-omic network for differentially regulated metabolic reactions in glycogen metabolism (A) and TCA cycle (B) in the liver and skeletal muscle. The information for glycogen metabolism and TCA cycle were obtained from “starch and sucrose metabolism” (mmu00500) or “TCA cycle” (mmu00020) in the KEGG database, respectively (Kanehisa et al., 2017). In Enzyme Protein layer of the trans-omic network for differentially regulated metabolic reactions in glycogen metabolism, we also added the information of phosphorylation of glycogen synthetase (pGs) and glycogen phosphorylase (pGp) (see also Figure 2A), which are key enzymes in the insulin signaling molecules that involved in the glycogen metabolism. Bar plots of measured molecules in WT and *ob*/*ob* mice are shown for corresponding nodes as the means and SEMs of mice replicate (number indicated in Transparent Methods). The colors of the frames indicate differentially expressed molecules increased in *ob*/*ob* mice (red) and those decreased in ob/ob mice (blue). The dashed frames indicate molecules that were not included in the trans-omic network of each organ. Diamond nodes indicate metabolic reactions. The colored edges indicate differential regulations: activating regulation in *ob*/*ob* mice (red) and inhibiting regulation in *ob*/*ob* mice (blue). From Metabolite to Metabolic Reaction, only allosteric regulations are colored. Black edges indicate the relationship between metabolic reactions and its substrate/product. The reversibility of metabolic reactions was obtained from the KEGG database (Kanehisa et al., 2017).

Glycogen is a glucose polymer stored in the liver and skeletal muscle, and the regulatory mechanism of glycogen metabolism is essential for blood glucose homeostasis. In the liver, glycogen and G6P significantly increased in *ob*/*ob* mice (Figure S6A left). The expression of enzyme proteins, such as Pgm1, Ugp2, Gys2, and Gbe1, which are responsible for a series of synthetic pathways from G6P to glycogen, increased in the liver of *ob*/*ob* mice. Pygb and Pygl, which catalyze the degradation of glycogen to G1P, also increased. Among the increased DEMEs in the liver of *ob*/*ob* mice, Pygb, Pygl, and Gbe1 showed the increased expression at the transcript level, and their gene expression was regulated by *Pparg*, *Esr1*, or *Klf4*. These results suggest that the turnover of glycogen synthesis and degradation increased in liver of *ob*/*ob* mice. A number of allosteric regulations by DEMs were also identified in the liver (8 activating and 14 inhibiting regulations in *ob*/*ob* mice). In contrast to glycogen metabolism in the liver, G6P [fold change (FC) (*ob*/*ob*/WT) = 0.66, q value = 0.13)] and glycogen [FC (*ob*/*ob*/WT) = 0.48, q value = 0.13] slightly decreased in skeletal muscle (Figure S6A right). Also, unlike the liver, there was no regulation in skeletal muscle from Enzyme Protein or Metabolite to the Metabolic Reaction, except for the activating regulation by phosphorylation of glycogen phosphorylase.

In the TCA cycle in the liver, citrate, cis-aconitate, and succinate increased in *ob*/*ob* mice (Figure S6B left). Malate [FC (*ob*/*ob*/WT) = 1.29, q value = 0.12] showed a slightly increased in the liver of *ob*/*ob* mice. In the regulations from Enzyme Protein to Metabolic Reaction in the liver, we identified the 9 activating and 6 inhibiting regulations in *ob*/*ob* mice. Cs, Acly, Aco1, and Aco2, which are responsible for metabolic reactions in the former half of the TCA cycle in the liver, increased in *ob*/*ob* mice, and indeed citrate and cis-Aconitate also increased in *ob*/*ob* mice. In addition, we identified many allosteric regulations by the increased DEMs in *ob*/*ob* mice (11 activating and 28 inhibiting regulations in ob/ob mice). Although no significant changes of amount of citrate and cis-Aconitate were detected between WT and *ob*/*ob* mice in skeletal muscle, citrate [FC (*ob*/*ob*/WT) = 1.16, q value = 0.18] and cis-Aconitate [FC (*ob*/*ob*/WT) = 1.27, q value = 0.24] shown slightly increases (Figure S6B right). We also identified Idh2, which is the increased DEME specifically in skeletal muscle. Furthermore, succinate and malate in the latter half of the TCA cycle showed a slightly increase in the liver of *ob*/*ob* mice, while those showed a slightly decrease in skeletal muscle of *ob*/*ob* mice (Figure S6B). Suclg2, a metabolic enzyme responsible for the conversion of succinyl-CoA to succinate, was the increased DEMEs common in both liver and skeletal muscle. The expression of *Suclg2* was upregulated at the transcript level both in the liver and skeletal muscle. *Suclg2* expression in the liver was regulated by *Pparg*, *Klf4*, and *Srebf1*, while that in skeletal muscle was regulated by *Ikzf1*, *Egr1*, and *Nfatc2*. In addition, succinate was identified as the increased DEM contributing to inhibiting regulations in the liver, while in skeletal muscle of *ob*/*ob* mice, it was identified as the decreased DEM contributing to activating regulations.

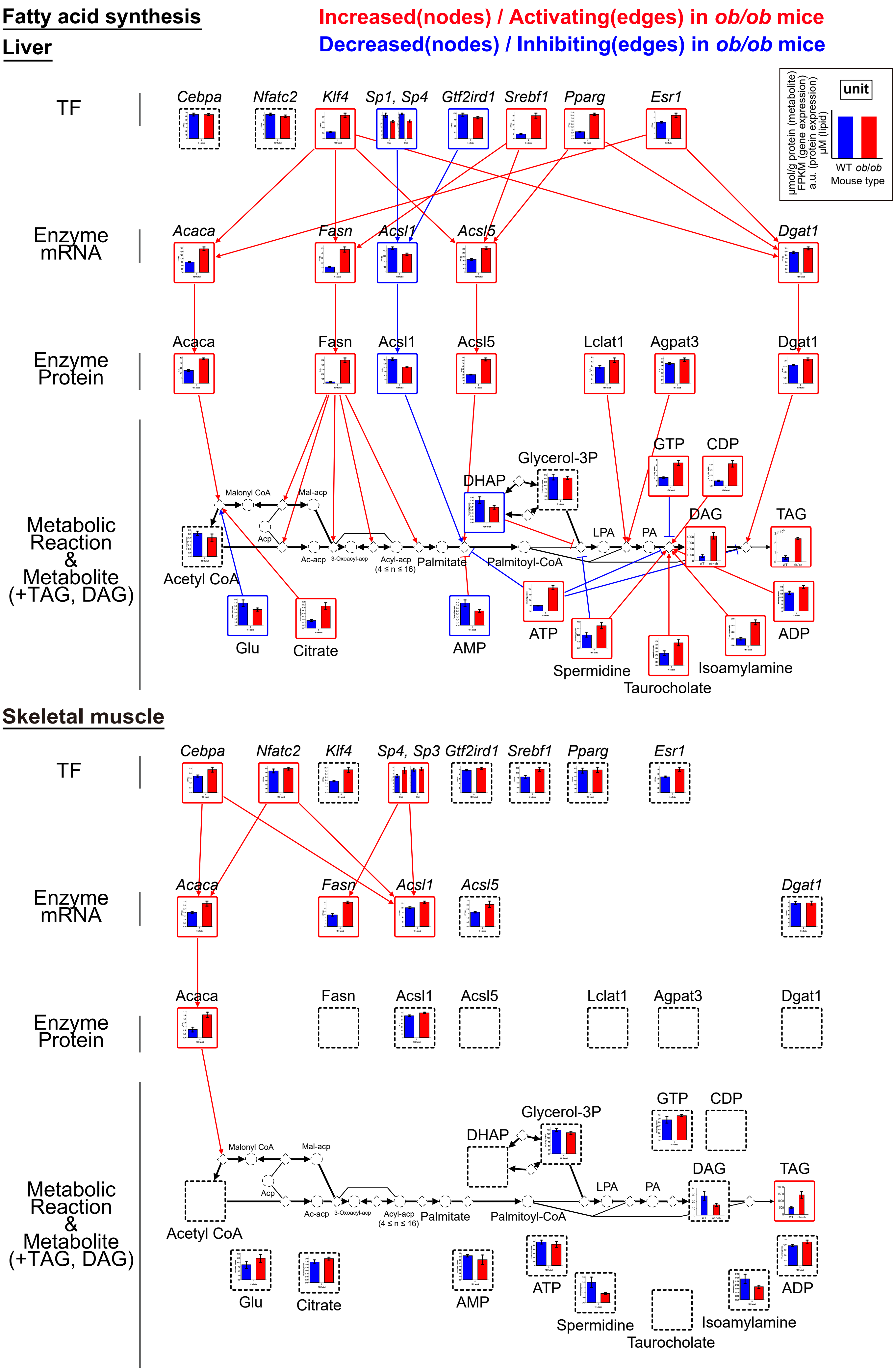

Fatty acid synthesis
Liver
Increased(nodes) / Activating(edges) in ob/ob mice
Decreased(nodes) / Inhibiting(edges) in ob/ob mice
unit
μmol/g protein (metabolite)
FPKM (gene expression)
a.u. (protein expression)
μM (lipid)
WT ob/ob
Mouse type
TF
Enzyme mRNA
Enzyme Protein
Metabolic Reaction & Metabolite (+TAG, DAG)
Cebpa
Nfatc2
Klf4
Sp1, Sp4
Gtf2ird1
Srebf1
Pparg
Esr1
Acaca
Fasn
Acsl1
Acsl5
Dgat1
Lclat1
Agpat3
Glycerol-3P
DHAP
GTP
CDP
Malonyl CoA
Mal-acp
Acp
Ac-acp
3-Oxoacyl-acp
Acyl-acp (4 ≤ n ≤ 16)
Palmitate
Palmitoyl-CoA
LPA
PA
DAG
TAG
Acetyl CoA
Glu
Citrate
AMP
ATP
Spermidine
Taurocholate
Isoamylamine
ADP
Skeletal muscle
Sp4, Sp3

**Figure S7. The Trans-Omic Network for Differentially Regulated Metabolic Reactions in Fatty Acid Synthesis. Related to Figure 4.**

The trans-omic network for differentially regulated metabolic reactions in fatty acid synthesis in the liver and skeletal muscle. The information for fatty acid synthesis were obtained from “fatty acid biosynthesis” (mmu00061), “glycerolipid metabolism” (mmu00561) and “glycerophospholipid metabolism” (mmu00564) in the KEGG database (Kanehisa et al., 2017). Data are shown as the mean and SEM of mice replicate (number indicated in Transparent Methods).

We investigated the fatty acid synthesis pathway (Figure S7) and fatty acid degradation pathway (see Figures 7A and 7B) in the liver and skeletal muscle, because fatty acid metabolism is a key factor in controlling lipid metabolic homeostasis. We identified a significant increase in Triacylglycerol (TAG) both in the liver and skeletal muscle of *ob*/*ob* mice in fatty acid synthesis pathways (Figure S7). Diacylglycerol (DAG), a precursor of TAG, also increased in the liver of *ob*/*ob* mice (Figure S7 upper). In the liver, we identified six increased DEMEs. The gene expression of *Acaca*, *Fasn*, *Acsl5*, and *Dgat1* was regulated by the increased DRTFs such as *Srebf1*, *Pparg*, *Esr1*, and *Klf4*. In addition, we identified several allosteric regulations by the DEMs in *ob*/*ob* mice (8 activating and 5 inhibiting regulations). Taken together, these results suggest that activation of fatty acid synthesis in the liver of *ob*/*ob* mice. In skeletal muscle of *ob*/*ob* mice, there was only activating regulations by the increased DEME Acaca (Figure S7 lower). Also, we were not able to detect any allosteric regulations by DEMs in skeletal muscle of *ob*/*ob* mice. Given that Acaca and TAG increased in skeletal muscle of *ob*/*ob* mice, this result implies that the fatty acid synthesis in skeletal muscle was also activated in skeletal muscle as well as in the liver. Note, however, that many of activating regulations in *ob*/*ob* mice found in the liver was not detected in skeletal muscle (Figure S7). In summary, fatty acid synthesis (Figure S7) and fatty acid degradation (Figures 7A and 7B) are commonly activated both in the liver and skeletal muscle of *ob*/*ob* mice. This result implies that fatty acid turnover may increase in both organs in obese individuals fasted for 16 hours.

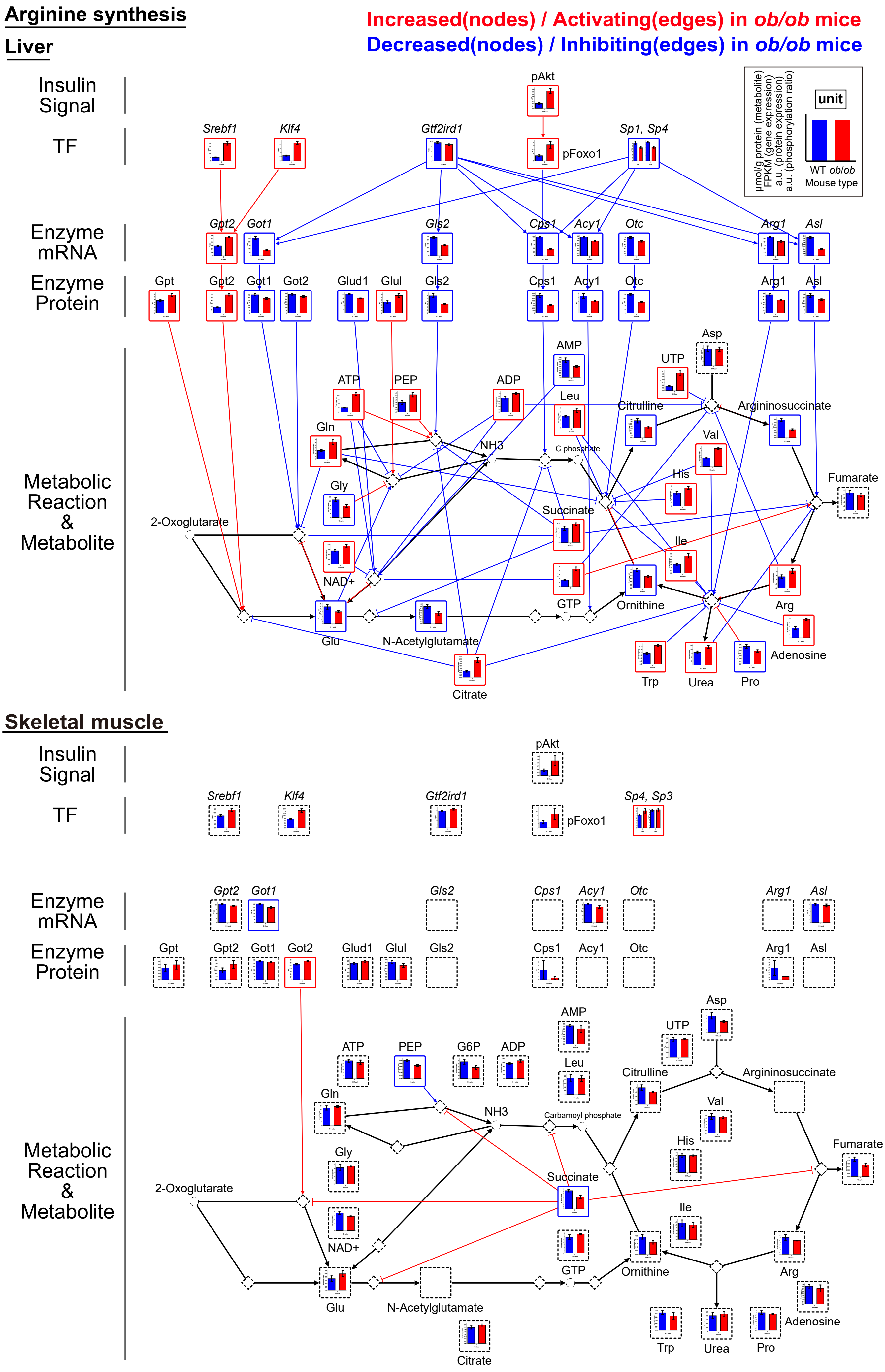
Arginine synthesis
Liver
Increased(nodes) / Activating(edges) in ob/ob mice
Decreased(nodes) / Inhibiting(edges) in ob/ob mice
unit
μmol/g protein (metabolite)
FPKM (gene expression)
a.u. (protein expression)
a.u. (phosphorylation ratio)
WT ob/ob
Mouse type
Insulin Signal
TF
Enzyme mRNA
Enzyme Protein
Metabolic Reaction & Metabolite
pAkt
pFoxo1
Srebf1
Klf4
Gtf2ird1
Sp1, Sp4
Gpt2
Got1
Gls2
Cps1
Acy1
Otc
Arg1
Asl
Gpt
Got2
Glud1
Glul
AMP
Asp
UTP
ATP
PEP
ADP
Leu
Citrulline
Argininosuccinate
Gln
NH3
C phosphate
Val
Gly
His
Fumarate
2-Oxoglutarate
Succinate
Ile
NAD+
GTP
Ornithine
Arg
Glu
N-Acetylglutamate
Adenosine
Trp
Urea
Pro
Citrate
Skeletal muscle
Insulin Signal
TF
pAkt
Srebf1
Klf4
Gtf2ird1
pFoxo1
Sp4, Sp3
Enzyme mRNA
Enzyme Protein
Gpt2
Got1
Gls2
Cps1
Acy1
Otc
Arg1
Asl
Gpt
Got2
Glud1
Glul
Metabolic Reaction & Metabolite
Asp
AMP
UTP
ATP
PEP
G6P
ADP
Leu
Citrulline
Argininosuccinate
Gln
NH3
Carbamoyl phosphate
Val
Gly
His
Fumarate
2-Oxoglutarate
Succinate
Ile
NAD+
GTP
Ornithine
Arg
Glu
N-Acetylglutamate
Adenosine
Trp
Urea
Pro
Citrate

**Figure S8. The Trans-Omic Network for Differentially Regulated Metabolic Reactions in Arginine Synthesis. Related to Figure 4.**

The trans-omic network for differentially regulated metabolic reactions in arginine synthesis in the liver and skeletal muscle. The information for arginine synthesis were obtained from "arginine biosynthesis" (mmu00220) in the KEGG database (Kanehisa et al., 2017). Data are shown as the mean and SEM of mice replicate (number indicated in Transparent Methods).

Arginine is synthesized mainly through the urea cycle. We investigated the arginine synthesis pathway in the liver, because the expression of genes encoding the urea cycle-related enzymes Cps1 and Otc decrease in obese patients with NASH and in obese mice fed a high fat and cholesterol diet (Chiara et al., 2018; Rodríguez-Suárez et al., 2010). We investigated arginine synthesis pathway also in skeletal muscle. We identified several decreased DEMs in the liver of *ob*/*ob* mice such as Glu, N-acetylglutamate, argininosuccinate, citrulline, and ornithine (Figure S8 upper). Gln, Arg, and Urea were the increased DEMs in the liver of *ob*/*ob* mice. In the liver, except for Gpt, Gpt2, and Glul, 9 DEMEs significantly decreased. Of the 9 decreased DEMEs in *ob*/*ob* mice, seven of them, including Cps1, Gls2, Otc, Arg1, and Asl, were regulated through changes in gene expression, and gene expression of them are regulated by some DRTFs such as *Gtf2ird1*, *Sp1*, *Sp4*, and phosphorylated Foxo1. Inhibition of transcriptional activity of Foxo1 by phosphorylation increased in ob/ob mice was conducted by increased Akt activation by phosphorylation. In addition, we identified many allosteric regulations by the DEMs (8 activating and 26 inhibiting regulations). These results suggest that the arginine synthesis in the liver is inhibited in *ob*/*ob* mice through the regulations by Enzyme Protein, which is consistent with previous reports, and by Metabolite. We identified the only DEME in skeletal muscle of *ob*/*ob* mice was identified as Got2, which was increased in *ob*/*ob* mice (Figure S8 lower). We examined the differential regulations by the DEMs in skeletal muscle, and identified those by PEP and succinate. Although many metabolites were measured in skeletal muscle, they did not show any changes in *ob*/*ob* mice compared to WT mice. All differentially expressed molecules in skeletal muscle in this pathway (Got2, succinate, and PEP) showed the opposite changes to the liver (Figure S8).

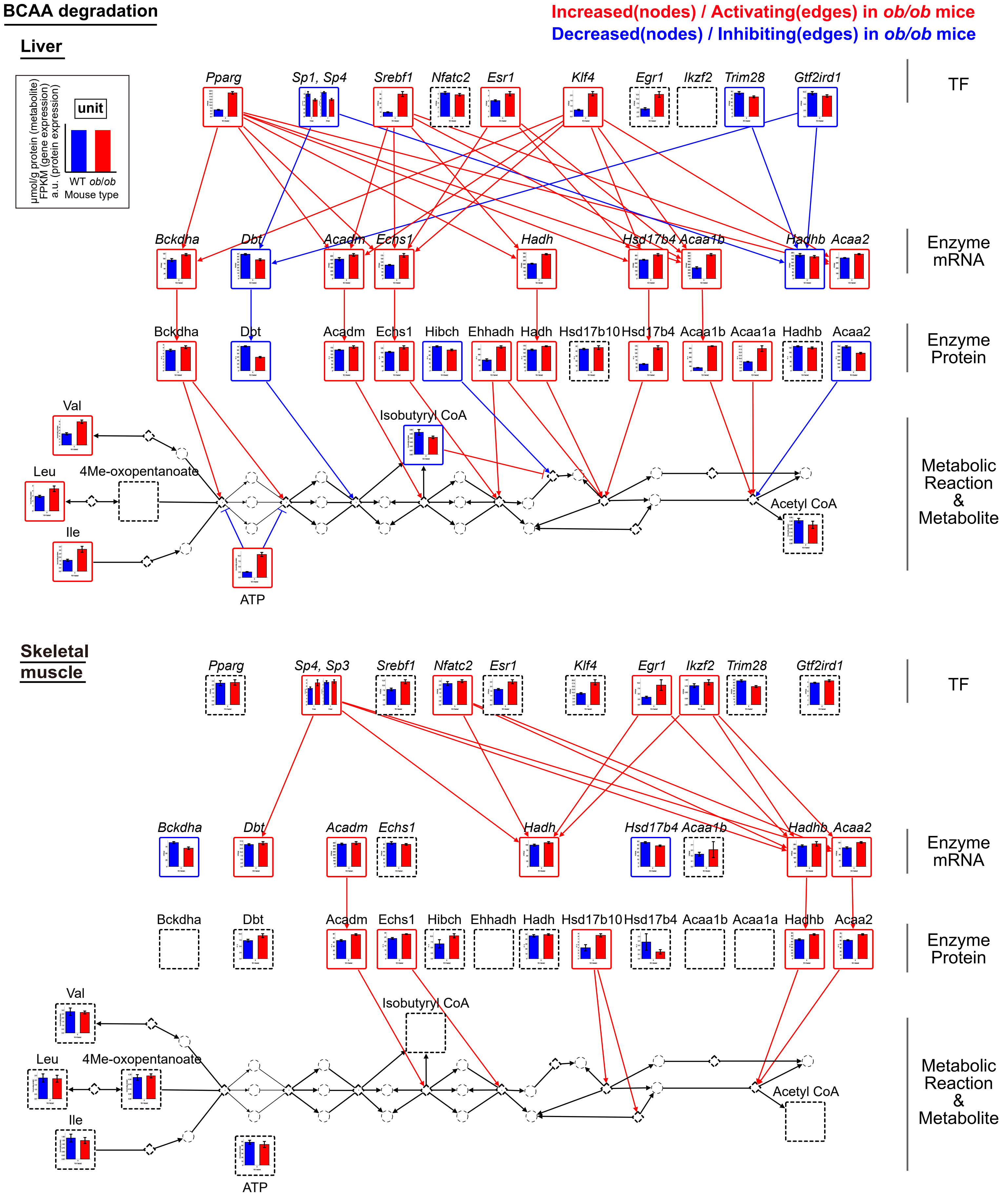
BCAA degradation
Increased(nodes) / Activating(edges) in ob/ob mice
Decreased(nodes) / Inhibiting(edges) in ob/ob mice
Liver
µmol/g protein (metabolite)
FPKM (gene expression)
a.u. (protein expression)
unit
WT ob/ob
Mouse type
TF
Enzyme mRNA
Enzyme Protein
Metabolic Reaction & Metabolite
Pparg
Sp1, Sp4
Srebf1
Nfatc2
Esr1
Klf4
Egr1
Ikzf2
Trim28
Gtf2ird1
Bckdha
Dbt
Acadm
Echs1
Hadh
Hsd17b4
Acaa1b
Hadhb
Acaa2
Hibch
Ehhadh
Hsd17b10
Acaa1a
Val
Leu
4Me-oxopentanoate
Ile
Isobutyryl CoA
Acetyl CoA
ATP
Skeletal muscle
Sp4, Sp3

**Figure S9. The Trans-Omic Network for Differentially Regulated Metabolic Reactions in BCAA Degradation. Related to Figure 4.**

The trans-omic network for differentially regulated metabolic reactions in BCAA degradation in the liver and skeletal muscle. The information for BCAA degradation were obtained from “valine, leucine and isoleucine degradation” (mmu00280) in the KEGG database (Kanehisa et al., 2017). Data are shown as the mean and SEM of mice replicate (number indicated in Transparent Methods).

BCAAs, three branched-chain amino acids among the essential amino acids, in organs and in the blood are known to be involved in insulin resistance associated with obesity and type 2 diabetes in several context (Newgard et al., 2009; White and Newgard, 2019). BCAAs are catabolized for energy source in several organs including of the liver, skeletal muscle, and adipose tissues. In the BCAA degradation pathway in the liver of *ob*/*ob* mice, we found increases of all BCAAs Val, Leu, and Ile (Figure S9 upper). Of the eight DEMEs whose expression increased in the liver of *ob*/*ob* mice, the expression of six proteins, including Bckdha, Acadm, Echs1, and Acaa1b, changed also at the transcript level, and those expression were upregulated by the increased DRTFs such as *Srebf1*, *Pparg*, *Esr1*, and *Klf4*. In addition, the increases of Acadm and Hadhb and Acaa2, among the five increased DEMEs, were seen also at the transcript level in skeletal muscle of *ob*/*ob* mice (Figure S9 lower). The expression of these genes was upregulated by *Sp4*, *Sp3*, *Nfatc2*, *Egr1*, and *Ikzf2*. Moreover, we found that Acadm and Ecsh1 increased common in the liver and skeletal muscle of *ob*/*ob* mice (Figure S9). These results suggest that in BCAA degradation pathway, the activations by DEMEs occurs both in the liver and skeletal muscle of *ob*/*ob* mice.

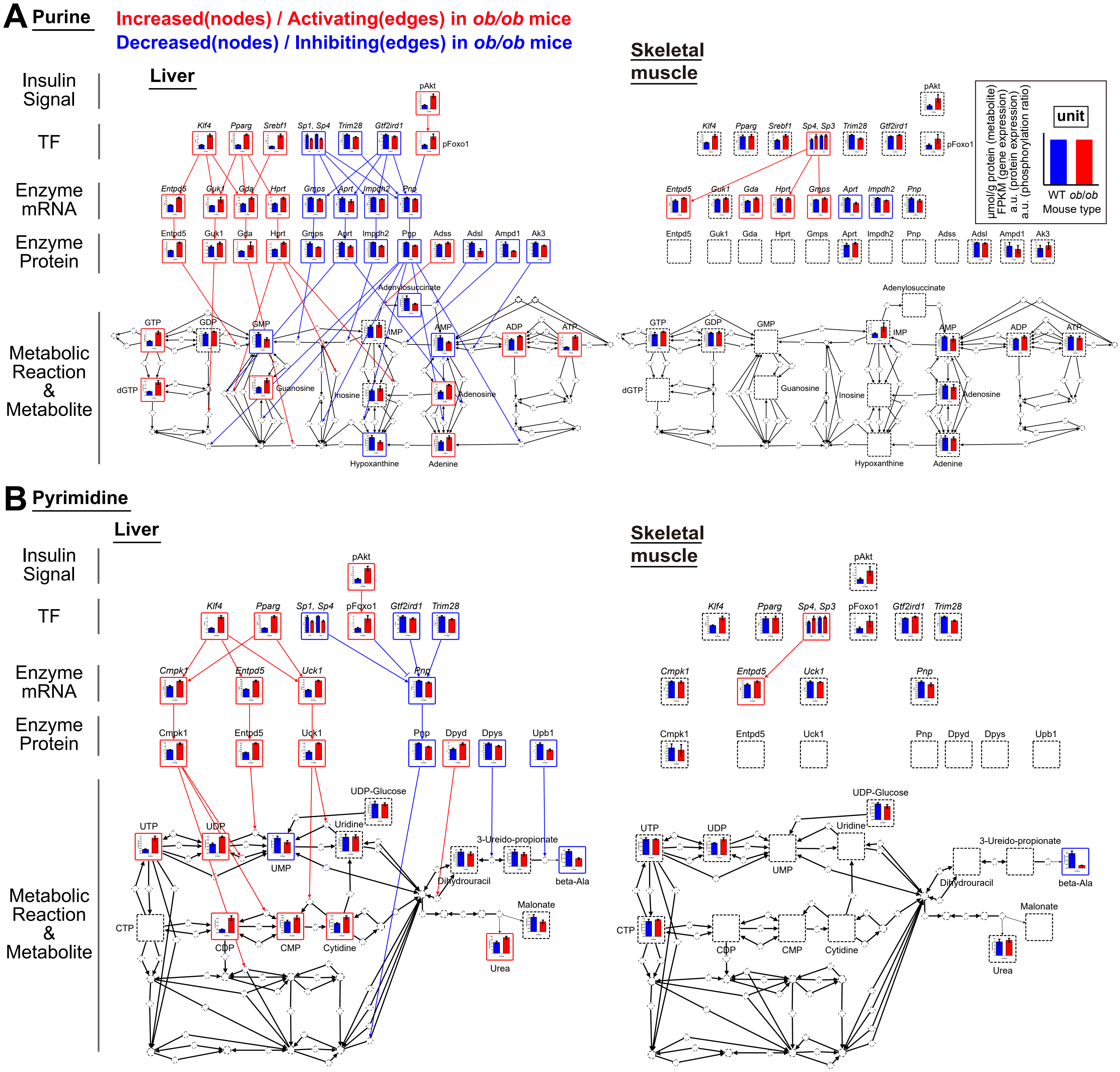
A Purine
Increased(nodes) / Activating(edges) in ob/ob mice
Decreased(nodes) / Inhibiting(edges) in ob/ob mice
Insulin Signal
TF
Enzyme mRNA
Enzyme Protein
Metabolic Reaction & Metabolite
Liver
Skeletal muscle
pAkt
Klf4
Pparg
Srebf1
Sp1, Sp4
Sp4, Sp3
Trim28
Gtf2ird1
pFoxo1
Entpd5
Guk1
Gda
Hprt
Gmps
Aprt
Impdh2
Pnp
Adss
Adsl
Ampd1
Ak3
Adenylosuccinate
GTP
GDP
GMP
IMP
AMP
ADP
ATP
dGTP
Guanosine
Inosine
Adenosine
Hypoxanthine
Adenine
unit
μmol/g protein (metabolite)
FPKM (gene expression)
a.u. (protein expression)
a.u. (phosphorylation ratio)
WT ob/ob
Mouse type
B Pyrimidine
Cmpk1
Uck1
Dpyd
Dpys
Upb1
UDP-Glucose
UTP
UDP
UMP
Uridine
CTP
CDP
CMP
Cytidine
3-Ureido-propionate
Dihydrouracil
beta-Ala
Malonate
Urea

**Figure S10. The Trans-Omic Network for Differentially Regulated Metabolic Reactions in Purine and Pyrimidine Metabolism. Related to Figure 4.**

(A, B) The trans-omic network for differentially regulated metabolic reactions in purine metabolism (A) and pyrimidine metabolism (B) in the liver and skeletal muscle. The information for purine metabolism or pyrimidine metabolism were obtained from "purine metabolism" (mmu00230) or "pyrimidine metabolism" (mmu00240) in the KEGG database, respectively (Kanehisa et al., 2017). Data are shown as the mean and SEM of mice replicate (number indicated in Transparent Methods).

In purine metabolism, the increases in GTP and ATP and the decreases in GMP and AMP were seen in the liver of *ob*/*ob* mice (Figure S10A left). We found that the expression of Entpd5, Guk1, Gda, and Hqrt increased in the liver of *ob*/*ob* mice, and gene expression of them was regulated by *Srebf1* and *Pparg*. In addition, among the 12 DEMEs, seven of those decreased in the liver of *ob*/*ob* mice. The decreased DEMEs in *ob*/*ob* mice contained Pnp, which contributes to the metabolic reaction of diverse nucleotide intermediates and whose expression at the transcript level was regulated by the DRTFs such as *Sp1*, *Sp4*, *Trim28*, *Gtf2ird1*, and phosphorylated Foxo1. In skeletal muscle, many nucleotides did not change between WT and *ob*/*ob* mice, except for a slightly increase in IMP [(fold change (*ob*/*ob*/WT) = 2.59, q value = 0.17)] (Figure S10A right).

In the pyrimidine metabolism, the increases in UTP, UDP, CDP, and CMP and the decreases in UMPs were found in the liver of *ob*/*ob* mice (Figure S10B left). The expression of metabolic enzymes surrounding the derivatives of Uridine and Cytidine, such as Cmpk1, Entpd5, and Uck1, were shown to increase in the liver of *ob*/*ob* mice. In addition, the expression of Dpys and Upb1, which are responsible for the series of metabolic reactions from dihydrouracil to beta-alanine in the liver, decreased in the liver of *ob*/*ob* mice. In skeletal muscle, there was no differential regulation by the expression changes of metabolic enzymes, and except for Cmpk1, no molecules were detected at the protein level (Figure S10B right). The beta-alanine was identified as the only DEM that decreased in common with both organs of *ob*/*ob* mice (Figure S10B).

To summarize the changes in metabolite amount in *ob*/*ob* mice, we found that dynamic changes of nucleic acid molecule were seen in the liver, whereas in skeletal muscle, those showed little changes. Triphosphate with three phosphate groups attached to ribose, such as GTP and ATP as derivatives of purines and UTP as derivatives of pyrimidines, significantly increased in the liver of *ob*/*ob* mice. In contrast, monophosphates such as GMP, AMP, and UMP significantly decreased in the liver of *ob*/*ob* mice.

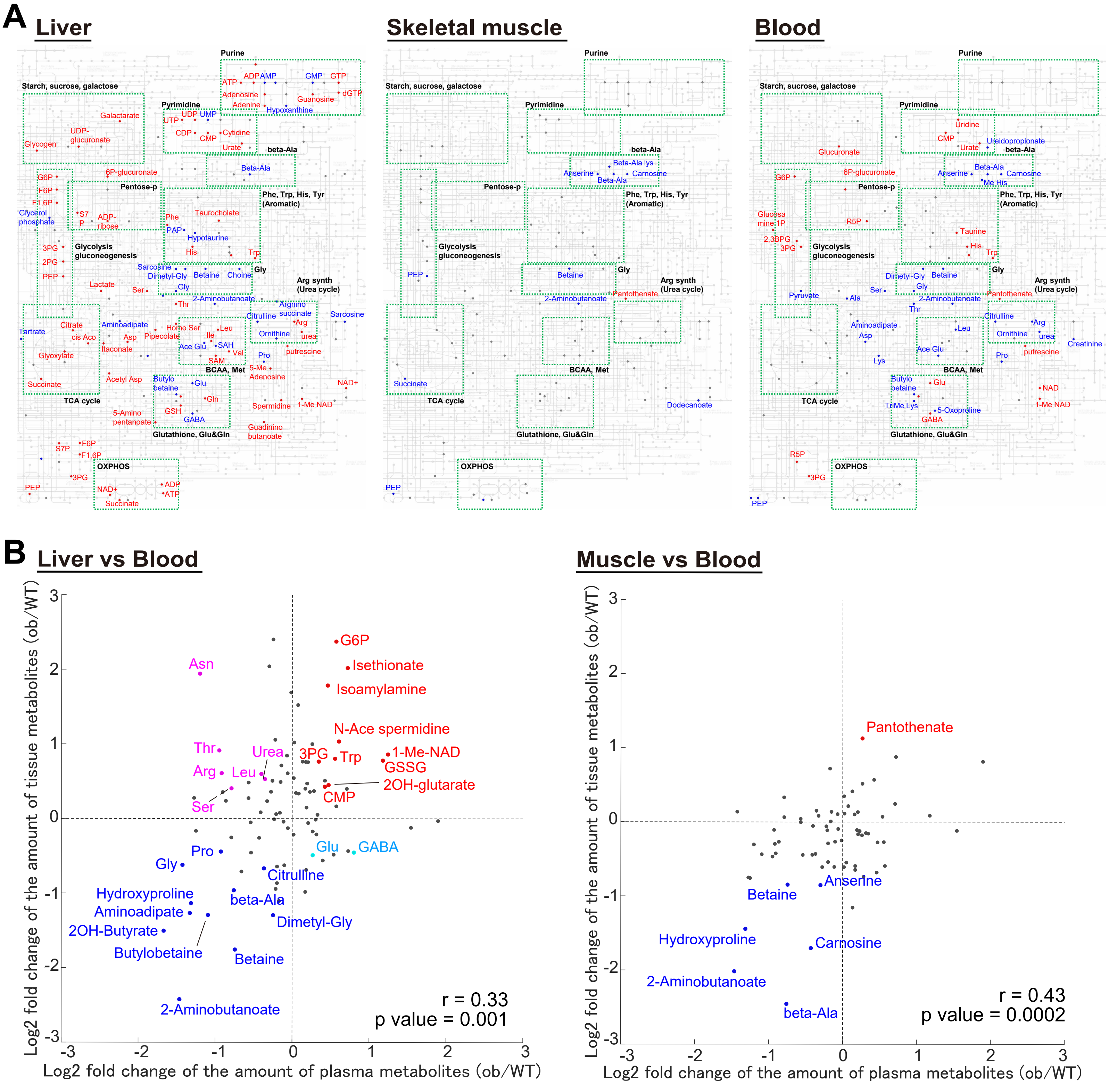


**Figure S11. Differentially Expressed Metabolites in Liver, Skeletal Muscle, and Blood. Related to Figure 5.**

(A) Information of metabolites of the liver, skeletal muscle, and blood projected on a global map of metabolic pathway (mmu01100) from the KEGG databases. Each dot on a global map was colored according to the variation of each metabolite: red dots, the increased DEMs in *ob*/*ob* mice; blue dots, the decreased DEMs in *ob*/*ob* mice; gray dots, those were measured in both WT and *ob*/*ob* mice but were not the DEMs.

(B) The $\log_2$ fold changes of metabolites measured in blood and each organ between WT and *ob*/*ob* mice. Each dot on a scatter plot was colored according to the variation of each metabolite: red dots, increased metabolites in *ob*/*ob* mice both in the organ and the blood; blue dots, decreased metabolites in *ob*/*ob* mice both in the organ and the blood; magenta dots, metabolites with increased in the organ and decreased in the blood in *ob*/*ob* mice; cyan dots, metabolites with decreased in the organ and increased in the blood in *ob*/*ob* mice.

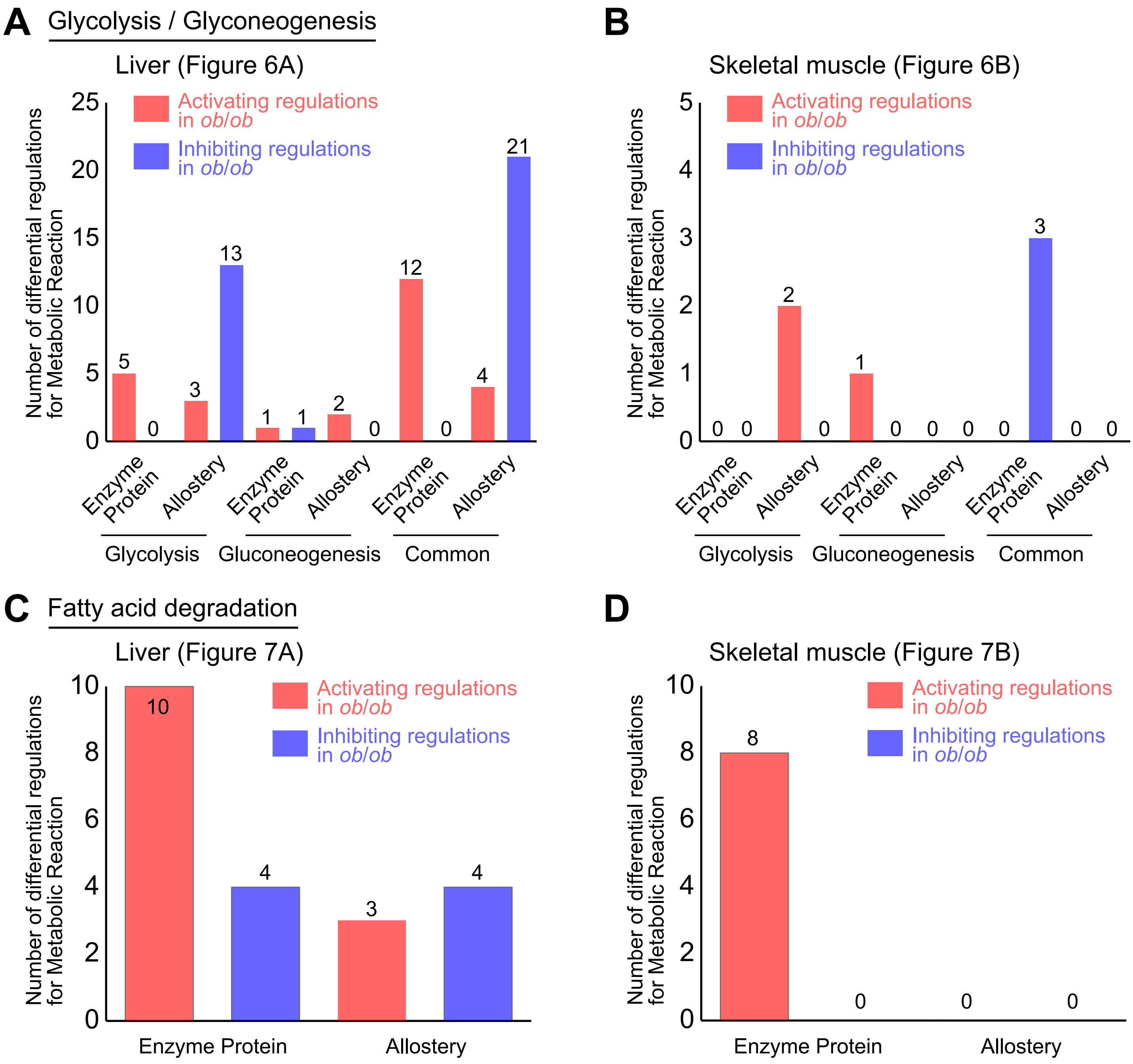


**Figure S12. The Activating and Inhibiting Differential Regulations for Metabolic Reaction of Trans-Omic Subnetworks. Related to Figures 6 and 7.**

The number of activating and inhibiting differential regulations of enzyme expression from Enzyme Protein layer and allosteric regulation from Metabolite layer in glycolysis/gluconeogenesis metabolic pathway in the liver (Figure 6A) (A) and skeletal muscle (Figure 6B) (B). The number of activating and inhibiting differential regulations of enzyme expression from Enzyme Protein layer and allosteric regulation from Metabolite layer in fatty acid degradation pathway in the liver (Figure 7A) (C) and skeletal muscle (Figure 7B) (D).

| | | Changes in our *ob*/*ob* mice | Changes in high-fat diet-fed mice (Soltis *et al*., 2017) |
|---|---|---|---|
| DETFs | *Srebf1* | Increase | Increase |
| | *Pparg* | Increase | Increase |
| | *Klf4* | Increase | Not change |
| | *Esr1* | Increase | Increase |
| | *Zfx* | Decrease | Decrease |
| | *Gtf2ird1* | Decrease | Decrease |
| | *Trim28* | Decrease | Decrease |
| | *Onecut1* | Decrease | Increase |
| | *Sp4* | Decrease | Not change |
| | *Sp1* | Decrease | Not change |

**Figure S13. Comparison of the Expression of DETFs in Liver of *ob*/*ob* Mice with those in Liver of High-Fat Diet-Fed Mice. Related to Figure 2 and Discussion.**

The table represents the expression changes of genes encoding to DETFs of our *ob*/*ob* mice in liver and those of high-fat diet-fed mice by Soltis *et al* (Soltis et al., 2017).

**TRANSPARENT METHODS**

Mouse Studies

Mouse experiments were approved by the animal ethics committee of The University of Tokyo. Ten-weeks-old male C57BL/6J wild-type (WT) and *ob*/*ob* mice were purchased from Japan SLC Inc. Sixteen hours-fasted mice were sacrificed by cervical dislocation, and the liver (whole or left lateral lobe) and skeletal muscle (gastrocnemius muscle) were dissected and frozen in liquid nitrogen. The frozen liver and skeletal muscle were pulverized with dry ice to a fine powder with a blender and separated into tubes for omic analyses (transcriptomics, proteomics, metabolomics, and lipidomics), and Western blotting. The following number of mouse replicates for each analysis was used: n = 5 both in WT and *ob*/*ob* mice for the identification of phosphorylated molecules by Western blotting, n = 11 (WT mice) and n = 12 (*ob*/*ob* mice) for transcriptomics, n = 5 both in WT and *ob*/*ob* mice for proteomics, n = 5 both in WT and *ob*/*ob* mice for metabolomics, n = 3 both in WT and *ob*/*ob* mice for lipidomics (see also Figure 1). The blood of each WT and *ob*/*ob* mice (n = 5) was collected from tail vein to measure blood glucose levels and insulin levels (see Figure S1). For blood metabolome analysis (see Figure 5), the blood of each WT and *ob*/*ob* mice (n = 5) was collected from the retro-orbital sinus into tubes containing 0.5 mg of EDTA soon after cervical dislocation, and the plasma was isolated by centrifugation at 2,300 × *g* for 15 min at 4ºC. We used the same data of the liver except for Western blotting analysis and the blood as we reported in our previous study (Kokaji et al., 2020). The skeletal muscle data were obtained from the same animals as the liver data. For Western blotting analysis, other individuals of mice were used.

Western Blotting

Total proteins were extracted from the liver and skeletal muscle with methanol:chloroform:water (2.5:2.5:1) extraction. Ice-cold methanol was added to the liver and skeletal muscle at a concentration of 100 mg/mL of the weight of the liver and skeletal muscle, and the suspension (400 µL) was mixed with chloroform (400 µL) and water (160 µL), followed by centrifugation at 4,600 × *g* for 10 min at 4ºC. Removing the aqueous and

organic phases, 800 μL of ice-cold methanol was added to the interphase to precipitate proteins. The obtained pellet was suspended with 400 μL of lysis buffer [10 mM Tris-HCl (pH 6.8) in 1% SDS], followed by sonication. The protein lysate was centrifuged at 12,000 × *g* for 3 min at 4ºC to remove debris. The concentration of total protein of the obtained supernatant was determined by BCA assay. Antibodies were purchased from Cell Signaling Technology, as follows: pIrβ (Tyr1150/Tyr1151) (#3024), total Irs1 (#2382), total Erk1/2 (#9102), pErk1/2 (Thr202/Tyr204) (#9101), total cAMP responsive element binding protein (Creb) (#9197), pCreb (Ser133) (#9198), total eukaryotic translation initiation factor 4e (eif4e) (#9742), peif4e (Ser209) (#9741), total Akt (#9272), pAkt (Ser473) (#9271), total S6 (#2217), pS6 (Ser235/Ser236) (#2211), total Gsk3β (#9315), pGsk3β (Ser9) (#9336), total Gs (#3886), pGs (Ser641) (#3891), total Ampkα (#2532), pAmpkα (Thr172) (#2531), total p38 (#9221), pp38 (Thr180/Tyr182) (#9211), total Foxo1 (#9462), pFoxo1 (Ser256) (#9461), and pAs160 (Thr642) (#8881). Antibodies against total Irβ (sc-711) and total Gp (sc-46347) were from Santa Cruz Biotechnology, and pIrs1 (Tyr612) (09-432), total Irs2 (MABS15) and total As160 (ABS54) were from Millipore. pGp (Ser15) was made in-house as previously described (Noguchi et al., 2013). The protein (20 μg or 30 μg) was separated on SDS-PAGE and blotted with the antibody. Immunodetection was carried out using Immobilon Western Chemiluminescent HRP Substrate (Millipore) or SuperSignal West Pico PLUS Chemiluminescent Substrate (Thermo Fisher Scientific), and the signals of Western blot were detected using a luminoimage analyzer (Fusion System Solo 7S; M&S Instruments Inc.), and quantified with the Fiji software (ImageJ; National Institutes of Health) (Schindelin et al., 2012). Contrast and brightness adjustment and treatment were performed with Photoshop CS6 (Adobe).

RNA Sequencing

Total RNAs were extracted from the liver and skeletal muscle using RNeasy Mini Kit (QIAGEN) and QIAshredder (QIAGEN) and assessed for quantity using Nanodrop (Thermo Fisher Scientific) and for quality using the 2100 Bioanalyzer (Agilent Technologies). cDNA libraries were generated using SureSelect strand-specific RNA library preparation kit (Agilent Technologies). The obtained cDNAs were subjected to 100-bp

paired-end sequencing on an Illumina HiSeq2500 Platform (Illumina) (Matsumoto et al., 2014). Sequences were aligned to the mouse reference genome obtained from the Ensembl database (Flicek et al., 2014; Cunningham et al., 2015) (GRCm38/mm10, Ensembl release 97) using the STAR software package (v.2.5.3a) (Dobin et al., 2013). To assemble transcript models (Ensembl release 97) from aligned sequences and to estimate gene expression level, the RSEM tool (v.1.3.0) was used (Li and Dewey, 2011). Gene expression level was represented as fragments per kilobase of exon per million mapped fragments (FPKM).

Proteomic Analysis

The liver and skeletal muscle were lysed with 0.5 mL of a solution containing 50 mM Tris-HCl pH8.8, 2%SDS, and 7 M urea, and then subjected to ultrasonic treatment with a Bioruptor (Digaenode). The samples were diluted with an equal volume of water and centrifuged at 15,000 *g* for 15 min at 4°C to remove insoluble fraction. The protein concentration of the lysates was determined with the bicinchoninic acid assay (Thermo Fisher Scientific) and adjusted to 1 mg/mL. Cysteine residues were blocked by incubation of the samples with 2 mM tris(2-carboxyethyl)phosphine hydrochloride (Thermo Fisher Scientific) for 30 min at 37 °C followed by alkylation with 10 mM 2-iodoacetamide for 30 min at room temperature. The proteins (200 μg) were precipitated with acetone for 3 hours at −30 °C and the resulting pellet was dispersed in 50 mM Triethylammonium bicarbonate by ultrasonic treatment (three times for 30 s with intervals of 30 s) with a Bioruptor (Diagenode). The protein suspension was subjected to digestion with lysyl endopeptidase (Wako) for 16 hours at 37 °C. Resulting peptides were centrifuged at 15,000 *g* for 15 min at 4°C and subjected to C18-StageTip purification prior to MS analysis (Rappsilber et al., 2007).

All samples were analyzed with Q Exactive (Thermo Fisher Scientific) instrument equipped with a Dionex Ultimate 3000 high-performance liquid chromatography (HPLC) System (Dionex Corporation) via a nano-electrospray source with a column oven set at 42 °C (AMR Inc.). Peptides were injected to pre-column, L-column micro (Chemicals Evaluation and Research Institute) and separated by in-house made 20 cm column filled with 2 μm octadecyl silane particle (Chemicals Evaluation and Research Institute) with a linear gradient

of 5–35% B for 110 min, 35–90% B for 1 min, and 90% B for 10 min at a flow rate of 200 nL/min, where A is 0.1% formic acid and B is 0.1% formic acid and 100% acetonitrile. Data acquisition was performed in data-dependent acquisition mode. Scan ranges were set at *m/z* 375-1600 for MS spectra and *m/z* 200-2000 for MS/MS spectra, respectively. MS spectra were acquired at a resolution of 70,000 at *m/z* 400 after accumulation to a target value of $1 \times 10^6$ with the maximum ion injection times for 60 msec. Up to the top 10, most abundant ions with charge 2+ or 3+ from the survey scan were selected with an isolation window of 1.5 *m/z* and then fragmented with an automatically optimized collision energy. MS/MS spectra were acquired at a resolution of 17,500 at *m/z* 400 after accumulation to a target value of $5 \times 10^4$ with the maximum ion injection times for 120 msec.

Raw data obtained from MS analyses were processed using MaxQuant software (version 1.6.0.16). MaxQuant uses its own search engine, andromeda, to search directly against the mouse Ensembl database (GRCm38/mm10, Ensembl release 97). The search was conducted with the following parameter settings: LysC/P, which also cleaves at carboxyl side of lysine, also if a proline follows, was selected as the enzyme used, the allowed number of missed cleavages was set to two, and carbamidomethylation of cysteine were selected as fixed modifications. Oxidized methionine was searched as variable modifications. Other parameters depended on the default settings in MaxQuant, which are optimized by the developers and are appropriate for most experiments.

<u>Metabolomic Analysis</u>

Total metabolites and proteins were extracted from the liver and skeletal muscle with methanol:chloroform:water (2.5:2.5:1) extraction. Approximately 40 mg of the liver and skeletal muscle was suspended with 500 µL of ice-cold methanol containing internal standards [20 µM L-methionine sulfone (Wako), 2-Morpholinoethanesulfonic acid (Dojindo), and D-Camphor-10-sulfonic acid (Wako)] for normalization of peak intensities of mass spectrometry (MS) among runs, then with, 500 µL of chloroform, and finally with 200 µL of water. After centrifugation at 4,600 × *g* for 15 min at 4ºC, the aqueous layer was filtered through a 5 kDa cutoff

filter (Human Metabolome Technologies) to remove protein contamination. The filtrate (320 μL) was lyophilized and, before MS analysis, dissolved in 50 μL water containing reference compounds [200 μM each of trimesate (Wako) and 3-aminopyrrolidine (Sigma-Aldrich)]. Proteins were precipitated by the addition of 800 μL of ice-cold methanol to the interphase and organic layers and separated by centrifugation at 12,000 × *g* for 15 min at 4ºC. The resulting pellet was washed with 1 mL of ice-cold 80% (v/v) methanol and resuspended in 1 mL of sample buffer containing 1% SDS and 50 mM Tris-Cl pH8.8, followed by sonication. The concentration of total protein was determined by BCA assay and was used for normalization of metabolite concentration among samples. Metabolites in the blood were extracted with methanol:chloroform:water (2.5:2.5:1). The blood (40 μL) was extracted with the sequential addition of 400 μL of ice-cold methanol containing the internal standards, 400 μL of chloroform, and 120 μL of water. After centrifugation at 10,000 × *g* for 3 min at 4 ºC, the aqueous layer was filtered through a 5 kDa cutoff filter (Human Metabolome Technologies) to remove protein contamination. The filtrate (300 μL) was lyophilized and, before analysis by MS, dissolved in 50 μL water containing the reference compounds.

All CE-MS experiments were carried out using an Agilent 1600 Capillary Electrophoresis system (Agilent technologies), an Agilent 6230 TOF LC/MS system, an Agilent 1200 series isocratic pump, a G1603A Agilent CE-MS adapter kit, and a G1607A Agilent CE electrospray ionization (ESI)-MS sprayer kit. Briefly, to analyze cationic compounds, a fused silica capillary [50 μm internal Diameter (i.d.) × 100 cm] was used with 1 M formic acid as the electrolyte (Soga and Heiger, 2000). Methanol/water (50% v/v) containing 0.01 μM hexakis(2,2-difluoroethoxy)phosphazene was delivered as the sheath liquid at 10 μL/min. ESI-TOFMS was carried out in positive ion mode, and the capillary voltage was set to 4 kV. Automatic recalibration of each acquired spectrum was achieved using the masses of the reference standards ([$^{13}C$ isotopic ion of a protonated methanol dimer $(2CH_3OH+H)]^+$, *m/z* 66.0631) and ([hexakis(2,2-difluoroethoxy)phosphazene +H]$^+$, *m/z* 622.0290). The metabolites were identified by comparing their m/z values and relative migration times to the metabolite standards. Quantification was performed by comparing peak areas to calibration curves generated using internal standardization techniques with methionine sulfone. The other conditions were identical to those

described previously (Soga et al., 2006). A commercially available COSMO(+) (chemically coated with cationic polymer) capillary (50 μm i.d. x 105 cm) (Nacalai Tesque, Kyoto, Japan) was used with a 50 mM ammonium acetate solution (pH 8.5) as the electrolyte to analyze anionic metabolites. Methanol/5 mM ammonium acetate (50% v/v) containing 0.01 μM hexakis(2,2-difluoroethoxy)phosphazene was delivered as the sheath liquid at 10 μL/min. ESI-TOFMS was carried out in negative ion mode, and the capillary voltage was set to 3.5 kV. Automatic recalibration of each acquired spectrum was achieved using the masses of the reference standards ([$^{13}$C isotopic ion of deprotonated acetate dimer ($2CH_3COOH$-H)]$^-$, *m/z* 120.0384) and ([hexakis(2,2-difluoroethoxy)phosphazene +deprotonated acetate ($CH_3COOH$-H)]$^-$, *m/z* 680.0355). D-Camphor-10-sulfonic acid was used as the internal standards for anion analysis. The other conditions were identical to those described previously (Soga et al., 2009). The acquired raw data were analyzed using our proprietary software (Sugimoto et al., 2010).

<u>Glycogen Content Assay</u>

Glycogen content assay was performed as previously described with some modifications (Noguchi et al., 2013). Approximately 20 mg of the liver and skeletal muscle was digested with 1.2 mL of 30% (w/v) potassium hydroxide solution for 1 hour at 95°C and neutralized with 61.2 μL of glacial acetic acid. The concentration of total protein of tissue digest was determined by BCA assay and adjusted to 1 μg protein/μL. Glycogen was extracted from the liver or skeletal muscle digest with Bligh and Dyer method to remove lipids. The tissue digest (50 μL) was mixed with 120 μL of ice-cold methanol, 50 μL of chloroform, 10 μL of 1% (w/v) linear polyacrylamide, and 70 μL of water. After incubation on ice for 30 min, the mixture was centrifuged at 12,000 × *g* to remove the aqueous layer. The glycogen was precipitated by the addition of 200 μL of methanol, separated by centrifugation at 12,000 × *g* for 30 min at 4ºC, washed with ice-cold 80% (v/v) methanol and dried thoroughly. The obtained glycogen pellets were suspended in 20 μL of 0.1 mg/mL amyloglucosidase (Sigma-Aldrich) in 50 mM sodium acetate buffer and incubated for 2 hours at 55ºC to digest glycogen.

According to the manufacturer's instruction, the glycogen-derived glucose concentration was determined using the Amplex Red Glucose/Glucose Oxidase Assay kit glucose assay (Thermo Fisher Scientific).

Lipidomic Analysis

The lipidomic profiling of the liver and skeletal muscle was performed by Metabolon, Inc. Lipids were extracted from samples using dichloromethane and methanol in a modified Bligh-Dyer extraction in the presence of internal standards with the lower, organic, phase being used for analysis. The extracts were concentrated under nitrogen and reconstituted in 0.25 mL of dichloromethane:methanol (50:50) containing 10 mM ammonium acetate. The extracts were placed in vials for infusion-MS analyses, performed on a SelexION equipped Sciex 5500 QTRAP using both positive and negative mode electrospray. Each sample was subjected to two analyses, with IMS-MS conditions optimized for lipid classes monitored in each analysis. The 5500 QTRAP was operated in MRM mode to monitor the transitions for over 1,100 lipids from up to 14 lipid classes. Individual lipid species were quantified based on the ratio of the signal intensity for target compounds to the signal intensity for an assigned internal standard of known concentration. Four-teen lipid class concentrations were calculated from the sum of all molecular species within a class.

Identification of Differentially Expressed Molecules between Wild-type Mice and *ob*/*ob* Mice

Molecules in the liver, skeletal muscle, and the blood, that were detected in less than 70% of replicates in either WT or *ob*/*ob* mice were removed from the analysis. The differences in the amounts of molecules between WT and *ob*/*ob* mice were determined by a statistical test using the following procedure. The significance of differences was tested by two-tailed Welch's t-test for each phosphorylation (ratio of phosphorylation to the total amount of molecule), protein expression, polar metabolite amount, and lipid amount, and by the edgeR package (version 3.29.1) of the R language (version 3.6.1) with default parameters for each gene expression. Molecules that showed a q value less than 0.1 for phosphorylation, protein expression, gene expression, polar metabolite amount, and lipid amount were defined as differentially expressed molecules in amount between

WT and *ob*/*ob* mice. The q values were calculated by Storey's procedure for gene expression, protein expression, and polar metabolite. The q values were calculated by Benjamini–Hochberg procedure for lipid and phosphorylation because of the small numbers of molecules (less than 100). The ratio of phosphorylation to the total amount of molecule was used to calculate fold change for all molecules.

Prediction of Transcription Factor Binding Motif and Inference of Differential Regulations between Differentially Regulated Transcription Factors and Differentially Expressed Genes

The flanking regions around the transcription start site of each DEG were extracted from GRCm38/mm10 (Ensembl, release 97) using Ensembl BioMart (Kinsella et al., 2011). The region from -1000 bp to +1000 bp of the major transcription start site was defined as the flanking region. The transcription factor (TF) binding motifs in each flanking region were predicted using TRANSFAC Pro, a TF binding motif database, and Match, a TF binding motif prediction tool (Matys et al., 2006; Kel et al., 2003). The threshold for each motif prediction was set using extended vertebrate_non_redundant_min_FP.prf, a TRANSFAC Pro parameter. Because some of the TFs known to involve the metabolic regulations of the liver or skeletal muscle are not included in this parameter set, we extracted the TF binding motifs of Pparα, Pparγ, Foxo1, Srebp1, and Chrebp1 from vertebrate_non_redundant.prf, and added these motifs and their parameters to vertebrate_non_redundant_min_FP.prf.

For the estimation of differential regulations between the regulating differentially regulated TFs (DRTFs) and the regulated DEGs, TF motif enrichment analysis was performed. The enrichment of the TF binding motif in the flanking regions of DEGs was determined by one-tailed Fisher's exact test. TF binding motifs with q value less than 0.05 were defined as significantly enriched. The q values were calculated by Benjamini–Hochberg procedure. The genes detected in more than 70% of the replicates in WT mice and *ob*/*ob* mice were used as a background. Of the TFs corresponding to enriched TF motifs, we identified those included in the DEGs as the differentially expressed TFs (DETFs) or in the DPPs as the differentially phosphorylated TFs (DPTFs), and defined them as the DRTFs. The differential regulations from the DRTFs to the DEGs were

determined based on the relationship between the TFs and the expression of the DEGs inferred from TF motif enrichment analysis. If a TF binding motif of DRTF was enriched in the flanking regions of the DEGs, the differential regulations between the DRTFs and the DEGs were inferred.

## Construction of the Trans-Omic Network for Differentially Regulated Metabolic Reactions in Liver and Skeletal Muscle

The trans-omic networks for differentially regulated metabolic reactions in liver and skeletal muscle consisted of differentially expressed molecules in six omic layers — Insulin Signal, TF, Enzyme mRNA, Enzyme Protein, Metabolic Reaction, and Metabolite layers— and the differential regulations connecting the differentially expressed molecules across each omic layer (see Figure 4A). The Insulin Signal layer is the insulin signaling pathway constructed in our previous study (Kawata et al., 2018; Kokaji et al., 2020). The Insulin Signal layer included insulin signaling molecules that we measured by Western blotting. The TF layer consisted of all TFs corresponding to inferred TF motifs (Table S6). The Enzyme mRNA layer and Enzyme Protein layer consisted of the expression of genes and proteins of all metabolic enzymes in the pathways in Metabolism obtained from the KEGG database (Kanehisa et al., 2017). The Metabolic Reaction layer consisted of the metabolic reactions (based on EC number) corresponding to the metabolic enzymes. The Metabolite layer consisted of all metabolites detected by CE-MS.

Each omic layer of the trans-omic network included the corresponding differentially expressed molecules. The Insulin Signal layer included insulin signaling molecules in differentially phosphorylated proteins (DPPs) (Figure 2A). The TF layer included DRTFs, which are TFs corresponding to inferred TF motifs included in the DEGs as the DETFs (Figure 2F) or those included in the DPPs as the DPTFs (Figure 2A). To avoid overestimation, the TFs with downstream genes that were not enriched in increased or decreased genes in *ob*/*ob* mice were excluded from DRTFs. The Enzyme mRNA layer included metabolic enzymes encoded by DEGs (Figure 2B). The Enzyme Protein layer included the differentially expressed metabolic enzymes (DEMEs), which were defined as metabolic enzymes among the differentially expressed proteins (DEPs)

(Figure 2C). The Metabolic Reaction layer included differentially regulated metabolic reactions, which were defined as metabolic reactions regulated by the differentially expressed metabolites (DEMs), the DEMEs, or both. The Metabolite layer included the DEMs (Figures 2D).

To determine differential regulations between the regulating differentially expressed molecules and the regulated differentially expressed molecules, we used a process that we have used previously (Kokaji et al., 2020). The differential regulations were determined using the changed directions (increased or decreased) of the regulating differentially expressed molecule, and the types of the regulatory directions (activator or inhibitor) of the regulating differentially expressed molecule (see also Figure S5 lower table). We defined the activating regulations in *ob*/*ob* mice when the regulating molecule increased in *ob*/*ob* mice was an activator or that decreased in *ob*/*ob* mice was an inhibitor and the regulated molecule increased in *ob*/*ob* mice. On the other hand, we defined the inhibiting regulations in *ob*/*ob* mice when the regulating molecule decreased in *ob*/*ob* mice was an activator or that increased in *ob*/*ob* mice was an inhibitor and the regulated molecule decreased in *ob*/*ob* mice.

The differential regulations in the trans-omic network from the Insulin Signal layer to the TF layer were determined based on the kinase-substrate relationship in the insulin signaling pathway constructed in our previous phosphoproteomic study (Kawata et al., 2019). The insulin signaling pathway comprises several signaling pathways in the KEGG database (Kanehisa et al., 2017). The differential regulations in the trans-omic network from the TF layer to the Enzyme mRNA layer were determined based on the relationship between DRTFs and the expression of DEGs inferred from TF motif enrichment analysis. According to the KEGG database, the effects of the phosphorylation of DRTFs on the types of regulations were defined. The differential regulations in the trans-omic network from the Enzyme mRNA layer to the Enzyme Protein layer were determined based on the correspondence between the DEGs encoding metabolic enzymes and the DEMEs. The differential regulations in the trans-omic network from the Enzyme Protein layer to the Metabolic Reaction layer were determined by regulation of metabolic reactions by the corresponding metabolic enzymes according to the KEGG database (Kanehisa et al., 2017). The differential regulations in the trans-omic network from the

Metabolite layer to the Metabolic Reaction layer were of two types: regulation by allosteric regulator among the DEMs (allosteric regulation) and regulation by the substrate or product among the DEMs. Allosteric regulations were determined by the regulation of metabolic reactions by allosteric regulators according to the BRENDA database (Jeske et al., 2019). The allosteric regulations reported for mammals (Bos taurus, Felis catus, Homo sapiens, “Macaca,” “Mammalia,” “Monkey,” Mus booduga, Mus musculus, Rattus norvegicus, Rattus rattus, Rattus sp., Sus scrofa, “dolphin,” and “hamster”) were used. According to the KEGG database, regulations by substrate or product were determined by the regulation of metabolic reaction by its substrate or product (Kanehisa et al., 2017). Because the reversibility of metabolic reactions was not determined, metabolic reactions were presumed to be regulated by both the substrate and product.

Implementation

Statistical tests and trans-omic network analysis were performed using MATLAB 2019a (The Mathworks Inc.). Visualization of trans-omic network in Graph Modeling Language (GML) formats was performed using Python 2.7 and VANTED (Junker et al., 2006).

**SUPLEMENTAL REFERENCES**

Chiara, F.D., Heebøll, S., Marrone, G., Montoliu, C., Hamilton-Dutoit, S., Ferrandez, A., Andreola, F., Rombouts, K., Grønbæk, H., Felipo, V., et al. (2018). Urea cycle dysregulation in non-alcoholic fatty liver disease. Journal of Hepatology *69*, 905–915.

Cunningham, F., Amode, M.R., Barrell, D., Beal, K., Billis, K., Brent, S., Carvalho-Silva, D., Clapham, P., Coates, G., Fitzgerald, S., et al. (2015). Ensembl 2015. Nucleic Acids Res *43*, D662–D669.

Dobin, A., Davis, C.A., Schlesinger, F., Drenkow, J., Zaleski, C., Jha, S., Batut, P., Chaisson, M., and Gingeras, T.R. (2013). STAR: ultrafast universal RNA-seq aligner. Bioinformatics *29*, 15–21.

Flicek, P., Amode, M.R., Barrell, D., Beal, K., Billis, K., Brent, S., Carvalho-Silva, D., Clapham, P., Coates, G., Fitzgerald, S., et al. (2014). Ensembl 2014. Nucleic Acids Res *42*, D749–D755.

Kinsella, R.J., Kähäri, A., Haider, S., Zamora, J., Proctor, G., Spudich, G., Almeida-King, J., Staines, D., Derwent, P., Kerhornou, A., et al. (2011). Ensembl BioMarts: a hub for data retrieval across taxonomic space. Database (Oxford) *2011*.

Li, B., and Dewey, C.N. (2011). RSEM: accurate transcript quantification from RNA-Seq data with or without a reference genome. BMC Bioinformatics *12*, 323.

Matsumoto, K., Suzuki, A., Wakaguri, H., Sugano, S., and Suzuki, Y. (2014). Construction of mate pair full-length cDNAs libraries and characterization of transcriptional start sites and termination sites. Nucleic Acids Res *42*, e125–e125.

Newgard, C.B., An, J., Bain, J.R., Muehlbauer, M.J., Stevens, R.D., Lien, L.F., Haqq, A.M., Shah, S.H., Arlotto, M., Slentz, C.A., et al. (2009). A Branched-Chain Amino Acid-Related Metabolic Signature that Differentiates Obese and Lean Humans and Contributes to Insulin Resistance. Cell Metabolism *9*, 311–326.

Noguchi, R., Kubota, H., Yugi, K., Toyoshima, Y., Komori, Y., Soga, T., and Kuroda, S. (2013). The selective control of glycolysis, gluconeogenesis and glycogenesis by temporal insulin patterns. Molecular Systems Biology *9*, 664.

Rappsilber, J., Mann, M., and Ishihama, Y. (2007). Protocol for micro-purification, enrichment, pre-fractionation and storage of peptides for proteomics using StageTips. Nature Protocols *2*, 1896–1906.

Rodríguez-Suárez, E., Duce, A.M., Caballería, J., Arrieta, F.M., Fernández, E., Gómara, C., Alkorta, N., Ariz, U., Martínez-Chantar, M.L., Lu, S.C., et al. (2010). Non-alcoholic fatty liver disease proteomics. PROTEOMICS – Clinical Applications *4*, 362–371.

Schindelin, J., Arganda-Carreras, I., Frise, E., Kaynig, V., Longair, M., Pietzsch, T., Preibisch, S., Rueden, C., Saalfeld, S., Schmid, B., et al. (2012). Fiji: an open-source platform for biological-image analysis. Nature Methods *9*, 676–682.

Soga, T., and Heiger, D.N. (2000). Amino Acid Analysis by Capillary Electrophoresis Electrospray Ionization Mass Spectrometry. Anal. Chem. *72*, 1236–1241.

Soga, T., Baran, R., Suematsu, M., Ueno, Y., Ikeda, S., Sakurakawa, T., Kakazu, Y., Ishikawa, T., Robert, M., Nishioka, T., et al. (2006). Differential Metabolomics Reveals Ophthalmic Acid as an Oxidative Stress Biomarker Indicating Hepatic Glutathione Consumption. J. Biol. Chem. *281*, 16768–16776.

Soga, T., Igarashi, K., Ito, C., Mizobuchi, K., Zimmermann, H.-P., and Tomita, M. (2009). Metabolomic Profiling of Anionic Metabolites by Capillary Electrophoresis Mass Spectrometry. Anal. Chem. *81*, 6165–6174.

Sugimoto, M., Wong, D.T., Hirayama, A., Soga, T., and Tomita, M. (2010). Capillary electrophoresis mass spectrometry-based saliva metabolomics identified oral, breast and pancreatic cancer-specific profiles. Metabolomics *6*, 78–95.

White, P.J., and Newgard, C.B. (2019). Branched-chain amino acids in disease. Science *363*, 582–583.
